# Weather-Resistant Thermoresponsive UV-Curable Smart Window Composites Based on Paraffin Particles

**DOI:** 10.1021/acsami.6c04877

**Published:** 2026-06-25

**Authors:** Marc Villabona, Lorenzo Vallan, Daniel Ruiz-Molina, Claudio Roscini, Jordi Hernando

**Affiliations:** † Departament de Química, Universitat Autònoma de Barcelona, Edifici C/n Campus UAB, Cerdanyola del Vallès 08193, Spain; ‡ Catalan Institute of Nanoscience and Nanotechnology (ICN2), CSIC and BIST, Campus UAB, Bellaterra 08193, Barcelona, Spain

**Keywords:** thermoresponsive smart windows, phase change materials, polymer composites, solid lipid particles, UV-curable coatings

## Abstract

Thermoresponsive composites made from phase change material particles embedded in polymer matrices show great promise for regulating sunlight transmittance in smart windows. However, their current performance is hindered by critical limitations. Composites obtained from water-soluble matrices present excellent optical performance, but they exhibit intrinsic poor weathering resistance, as they swell and dissolve upon water exposure (e.g., during rainfall or cleaning with aqueous solutions). In contrast, those prepared from water-resistant curable polymers typically require expensive or custom-made phase change materials, and very often show undesired optical properties (e.g., opaque states at room temperature or below). To address this challenge, in this work we developed novel thermoresponsive composite films comprising readily available paraffin particles dispersed within a cross-linked, water-insoluble acrylate matrix. The resulting materials exhibit excellent resistance to water while preserving the characteristic smart window behavior of paraffin-polymer composites; i.e., they remain transparent at low temperatures due to refractive index matching between the matrix and the solid paraffin particles, and become opaque once this condition is lost upon thermally induced paraffin melting, enabling efficient modulation of solar heating gain. In addition, because paraffin-acrylate films are produced via photopolymerization, they are obtained in a fast, straightforward and scalable manner. These features, combined with their low cost, mechanical flexibility, and multistimuli-responsive behavior, make paraffin-acrylate composites a robust and scalable platform for next-generation smart window technologies.

## Graphical abstract

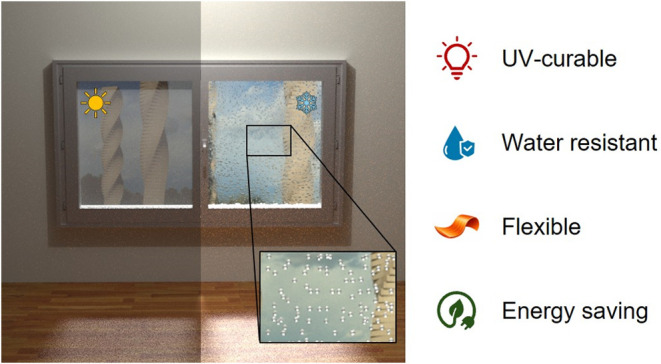

## Introduction

Buildings represent a significant share of global energy consumption, accounting for roughly 40% of total energy use in developed countries.^1–3^ Nearly half of this demand originates from lighting, heating, ventilation, and air conditioning systems, and it is therefore strongly related to light and heat transfer through windows.^1,3^ For this reason, one of the most promising strategies proposed for enhancing building energy efficiency is the adoption of smart windows (SWs).^4–9^ These advanced, stimuli-responsive fenestration elements dynamically adjust their solar light transmission based on outdoor conditions; i.e., they remain highly transparent during cold or cloudy days to maximize natural light and passive heating, while reducing sunlight penetration in warm weather to limit cooling energy requirements. Moreover, SWs contribute to enhancing occupant visual comfort and ensuring privacy within buildings, while also serving in applications that require precise regulation of solar irradiation, such as in greenhouses.^10,11^

Among the various types of SW technologies developed to date, thermoresponsive smart windows (TSWs) are particularly attractive because they enable passive modulation of solar transmittance in response to temperature fluctuations, thereby automatically adapting to changing weather conditions without requiring additional energy use.^4,7,12^ Typically, TSWs rely on organic or inorganic materials that undergo a temperature-driven phase transition, resulting in a variation of light absorption or scattering. This is the case of copolymers,^13^ hydrogels,^14–17^ ionogels,^18–20^ liquid crystals,^21–23^ and vanadium oxides,^24–26^ which are the most widely investigated systems for TSWs. However, the resulting devices exhibit several limitations, such as the high cost of their active components (e.g., liquid crystals and ionic liquids), their complex water-containing architectures (e.g., hydrogels), and the limited tunability of their thermal response (e.g., vanadium oxides).

To address these challenges, we^11,27^ and others^28,29^ have recently introduced a new type of phase change thermochromic material for TSW construction: composite films comprising a polymer layer loaded with thermoresponsive paraffin particles. At temperatures below paraffin melting point (*T*_m_^paraffin^), these composites remain highly transparent due to refractive index matching between the solid particles and the surrounding matrix. When heating above *T*_m_^paraffin^ to induce paraffin melting, this condition is lost, leading to increased light scattering at the particle–polymer interface, which turns the composite material opaque and efficiently blocks solar radiation for particles with sizes of hundreds of nanometers (>150 nm). Notably, this thermoinduced modulation of sunlight transmittance is accompanied by a number of additional advantages that position paraffin-based polymer composites as a highly promising TSW technology:^11,27–29^ modulation of both visible and near-infrared (NIR) light; low cost (at least an order of magnitude lower than current commercial SWs); thin, flexible structure that can be adhered onto preexisting windows; facile tunability of thermal response through paraffin composition variation; multimodal responsiveness to solar irradiation intensity and external electrical stimuli via photo- and electrothermal effects; compatibility with other SW technologies such as vanadium oxide-based TSWs; and enhanced thermal management through the latent heat associated with paraffin melting.

Despite these numerous advantages, the paraffin-based TSW films and coatings reported to date suffer from a critical drawback: they are not resistant to direct water exposure (e.g., cleaning with aqueous solutions), nor are they suitable for outdoor conditions such as rain or humid environments. The reason is that these materials are formulated from water-soluble polymers, such as poly­(vinyl alcohol) (PVA)^11,27,28^ or hydroxypropyl methylcellulose (HPMC),^29^ to warrant the required immiscibility with the paraffin particles for its processability from aqueous slurries. As a result, most of these materials also suffer from tedious preparation through casting-based methods that require slow evaporation of aqueous paraffin-polymer formulations,^11,27,28^ a drawback that could be surpassed with faster fabrication methods such as *in situ* photopolymerization. To address these challenges, water-resistant lipophilic UV- or thermally curable cross-linked polymers embedding phase segregated paraffin particles could be employed, as reported for liquid crystal-based TSWs.^30,31^ However, attempts to realize TSW films from paraffins^32^ or other low cost phase change materials such as ethylene glycol^33^ following this strategy have so far been unsuccessful, yielding composite films with undesired optical behavior for smart window applications―i.e., blocking sunlight at (or below) room temperature.^32,33^ Therefore, the fabrication of water-stable TSW coatings based on cost-effective paraffin materials and fast processing methods that reliably exhibit the required transparent-to-opaque switching upon thermally induced solid-to-liquid transition still remains a challenge.

Herein we successfully overcame this challenge by using commercially available, UV-curable acrylate-based monomers, which offer two major advantages for composite preparation: (a) their polarity can be tuned as to warrant immiscibility with paraffin droplets and particles, ensuring sufficient colloidal stability along the fabrication process; and (b) they produce highly cross-linked matrices, providing excellent mechanical and water resistance while exhibiting good refractive index matching with solid paraffins. To exploit these features, 2-hydroxyethyl methacrylate (HEMA) and poly­(ethylene glycol) dimethacrylate (PEGDA, number-average molecular weight ∼700 g mol^–1^) were selected as mono- and difunctional acrylate monomers, respectively, because they are oleophobic and can be photopolymerized under UV irradiation, yielding solid transparent films with refractive index values (RI) similar to those of solid paraffins (RI ∼ 1.51, Figure 1a).

**Figure 1:**
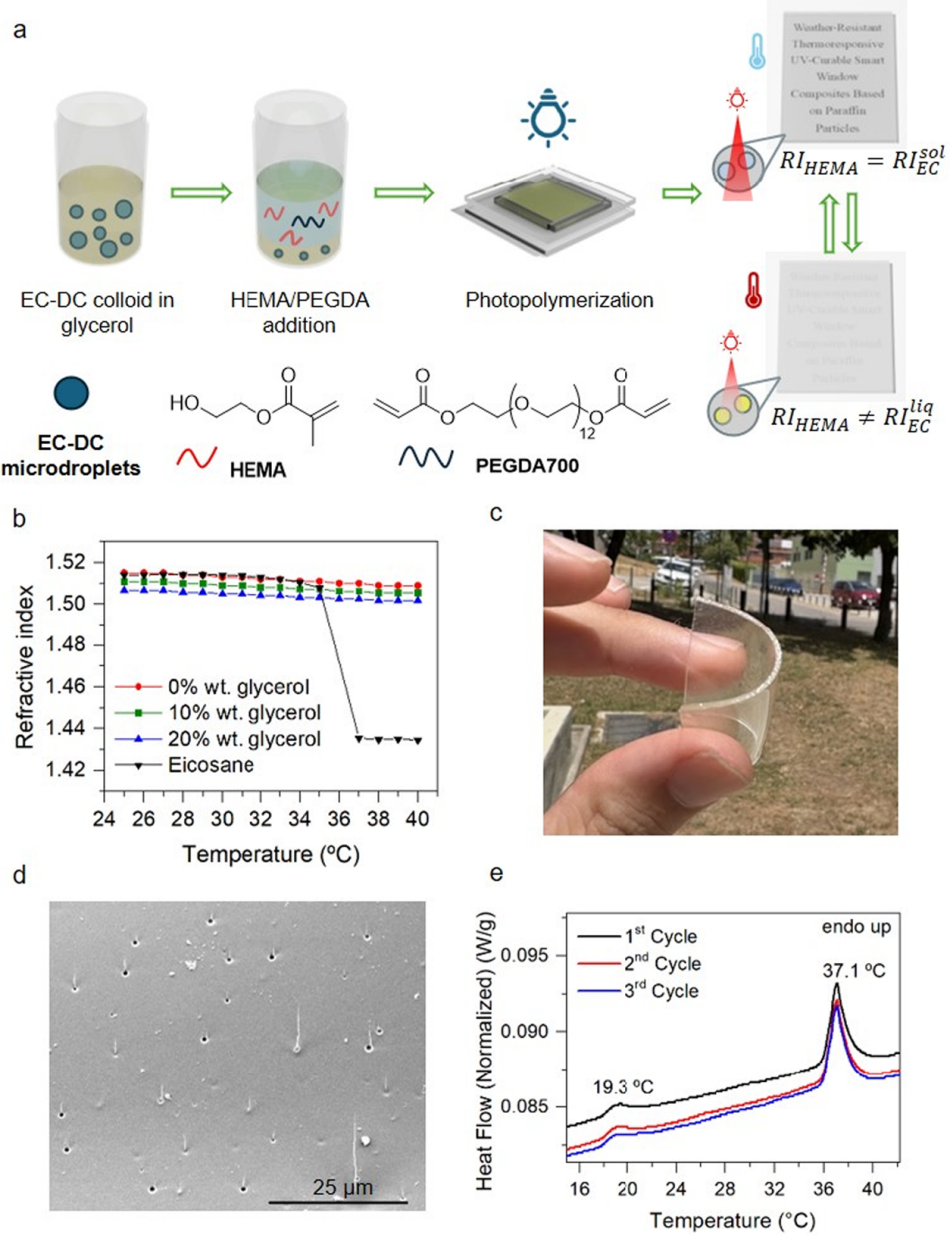
(a) Strategy used in this work for the preparation of paraffin-acrylate composites exhibiting reversible, thermally induced transparent-to-opaque modulation upon temperature variation. (b) Temperature variation of the refractive index of bulk eicosane and of solid HEMA-PEGDA films with different glycerol content (0% wt., 10% wt., 20% wt.). (c) Photograph of a flexible paraffin-acrylate composite film (0.4% wt. EC-DC particles, 19.6% wt. glycerol, 72% wt. HEMA, 8% wt. PEGDA) at room temperature. (d) SEM image of the cross-section of the paraffin-acrylate composite film shown in (c) after treatment with hexane to dissolve the EC-DC particles exposed at the surface. (e) Endothermic region of 3 consecutive DSC thermograms measured for the paraffin-acrylate composite.

## Results and Discussion

### Preparation of Water-Resistant Thermochromic Paraffin-Polymer Composites

To obtain thermochromic paraffin-acrylate composite films from HEMA and PEGDA, we used paraffin particles made of a 9:1 mixture of eicosane (EC) and docosane (DC). As we have recently reported,^11^ these mixed EC-DC particles provide optimal solar transmittance modulation when dispersed within PVA films, which occurs around the melting temperature of its main paraffinic component (*T*_m_^EC^ = 37 °C).^34^ However, the formation of stable dispersions of these paraffin particles with HEMA and PEGDA turned out to be challenging. When melted at the temperatures reached during the emulsification process required for colloid preparation, EC was found to be slightly miscible with these acrylate monomers, which prevented direct obtention of dispersions of EC-DC particles in liquid HEMA and PEGDA. To circumvent this issue, the more polar glycerol was instead used as the liquid medium for emulsification, which was conducted by ultrasonication at high temperatures. Subsequent cooling of the resulting emulsion ensured crystallization of the liquid EC-DC droplets into solid particles, thereby producing the desired colloidal dispersion. By tuning the amount of paraffins and glycerol, this colloid sustained posterior addition of HEMA and PEGDA, finally obtaining a polymerizable mixture that could be used for the preparation of paraffin-acrylate composites (Figure 1a and Table S1). Although this implied that small amounts of glycerol (RI = 1.473) would remain dispersed within these composites after polymerization, minimal effects were observed on the refractive index matching conditions required for TSW operation (Figure 1b).

To produce paraffin-acrylate composites from the mixtures of EC-DC particles, HEMA, PEGDA and glycerol, the syrup was first introduced between two glass substrates, separated by double-sided tape used as a spacer to control film thickness (Figure S1a), and then photopolymerized. Several radical photoinitiators and UV illumination conditions were tested, obtaining the best results with 2,2-dimethoxy-2-phenylacetophenone (DMPA) and 60 s irradiation at 365 nm (Table S2). Subsequent detachment from glass yielded self-standing cross-linked acrylate films, with exhibited homogeneous and reproducible thickness of ca. 1.0 mm (Figure S1b). Because of its low volatility (boiling point: 290 °C),^34^ all glycerol used for formulation preparation remains in the final photopolymerized composites, providing good mechanical flexibility thanks to its plasticizing effect (Figure 1c), as previously reported by us for paraffin-PVA films.^11,27^ Interestingly, this methodology enables the preparation of thermoresponsive composites in very short times (<120 s), in sharp contrast to previous procedures that rely on casting aqueous dispersion of paraffin particles, which required from 30 min to several hours to ensure water evaporation and film formation.^11,27^

The presence of EC-DC particles within the photopolymerized films was corroborated by both scanning electron microscopy (SEM) and differential scanning calorimetry (DSC) measurements. SEM analysis was performed on cross sections of the films that had been previously treated with hexane to selectively dissolve the paraffin particles exposed at the surface. As a result, voids were generated in the material that could be imaged and characterized by SEM, exhibiting an average diameter of 730 nm and a broad size distribution mainly ranging from 75 to 1500 nm (Figures 1d and S2). This result demonstrates that photopolymerization preserved the integrity and dispersion of the embedded EC-DC particles. More importantly, it proves that their sizes fall within the range required to ensure ample modulation of sunlight scattering upon melting, as established on our previous works on paraffin-PVA composites.^11,27^ In these precedents we identified three different optical behaviors depending on particle diameter: (a) for very small particles (tens of nanometers in diameter), minimal visible light scattering is caused by both solid- and liquid-state particles, irrespective of the refractive index difference with the surrounding polymer matrix, leading to high transparency across all temperatures that could be exploited in other photonic applications (e.g., reconfigurable 2D lenses);^27,35^ (b) for large particles with diameters of a few micrometers, increased sunlight scattering is observed even at temperatures below paraffin melting, making the resulting composite films opaque over the whole thermal range;^27^ and (c) for intermediate-sized particles of several hundreds of nanometers in diameter, a large variation in solar light scattering and, therefore, transmittance is achieved upon paraffin phase change, reaching the desired transparency modulation for smart window applications.^11,27^ Actually, using Mie theory^36^ for EC-DC particles with a diameter of 730 nm, we calculated a pronounced change in the scattering cross-section in the visible region upon paraffin melting within the acrylate layers (σ_scattering_^550nm^ = 876.8 and 5.740·10^4^ nm^2^ at 25 and 40 °C, respectively), which should result in strong modulation of visible light transmittance even at low particle concentration (e.g., %Tr_550nm_ = 90.8 and 0.2% at 25 and 40 °C for 1 mm-thick films with 0.4% wt. of EC-DC particle concentration, see Supporting Information). On the other hand, we also proved by DSC measurements that the thermal properties of EC-DC particles are maintained within the cross-linked acrylate films (Figures 1e and S3). When heating, small but visible endothermic peaks were observed in the DSC thermogram of the paraffin-polymer composites that are characteristic of mixed EC-DC 9:1 particles:^11^ one peak at around 37 °C, which can be ascribed to melting, and another peak at about 19 °C, which corresponds to the transition between two different solid phases with similar refractive indices.

Once identified the conditions to produce paraffin-acrylate films by photopolymerization, further experiments were conducted to fine-tune their composition to achieve optimal TSW behavior. Thus, different amounts of glycerol dispersions of EC-DC particles, HEMA and PEGDA were mixed and polymerized, and their transmittance spectra were measured at room temperature and after paraffin melting at 40 °C to assess their capacity to thermally modulate sunlight transmission (Table S3). In these experiments, the incorporation of additives to enhance the refractive index matching between the cross-linked acrylate matrix and the solid paraffin particles was also considered, such as poly­(2-hydroxyethyl methacrylate) or poly­(methyl methacrylate). After preliminary experimental screening, the best results were obtained for composites comprising 0.4% wt. EC-DC particles, 19.6% wt. glycerol, 72% wt. HEMA, and 8% wt. PEGDA. At room temperature, these materials showed good optical transparency (∼80%) across the visible range and part of the near-infrared region (390–1300 nm), a spectral window that covers most solar irradiance spectrum (Figure 2a,b). In particular, the transmittance in the visible or luminous range (λ_lum_ = 390–780 nm) at 24 °C was found to be % Tr_lum_^24°C^ = 78%, which closely matches the figures accomplished with paraffin-PVA composites (% Tr_lum_^24°C^ = 70–80% ^11,27^ ). On the other hand, our paraffin-acrylate films turned opaque upon heating above the melting temperature of EC-DC particles (e.g., 40 °C, Figure 2a,b). For the entire solar irradiance spectrum, we determined a thermal change in transmittance of % ΔTr_solar_^24–40°C^ = 35%, with larger modulation achieved in the visible region (% ΔTr_lum_^24–40°C^ = 43%) than in the near-infrared spectrum (% ΔTr_NIR_^24–40°C^ = 25%). These values fall slightly below those reported for paraffin-PVA composites (ΔTr_solar_^24–40°C^ = 42–59% ^11,27^ ) and deviate from the simulations conducted using Mie scattering theory for composites bearing uniform 730 nm-in-diameter paraffin particles (% ΔTr_550nm_^25–40°C^ = 90%). The latter might be ascribed to the size distribution of the actual particles used in the paraffin-acrylate films, as those with diameters smaller than 730 nm (ca. 48%) will produce less solar light scattering, reducing the transparency modulation upon melting.

**Figure 2:**
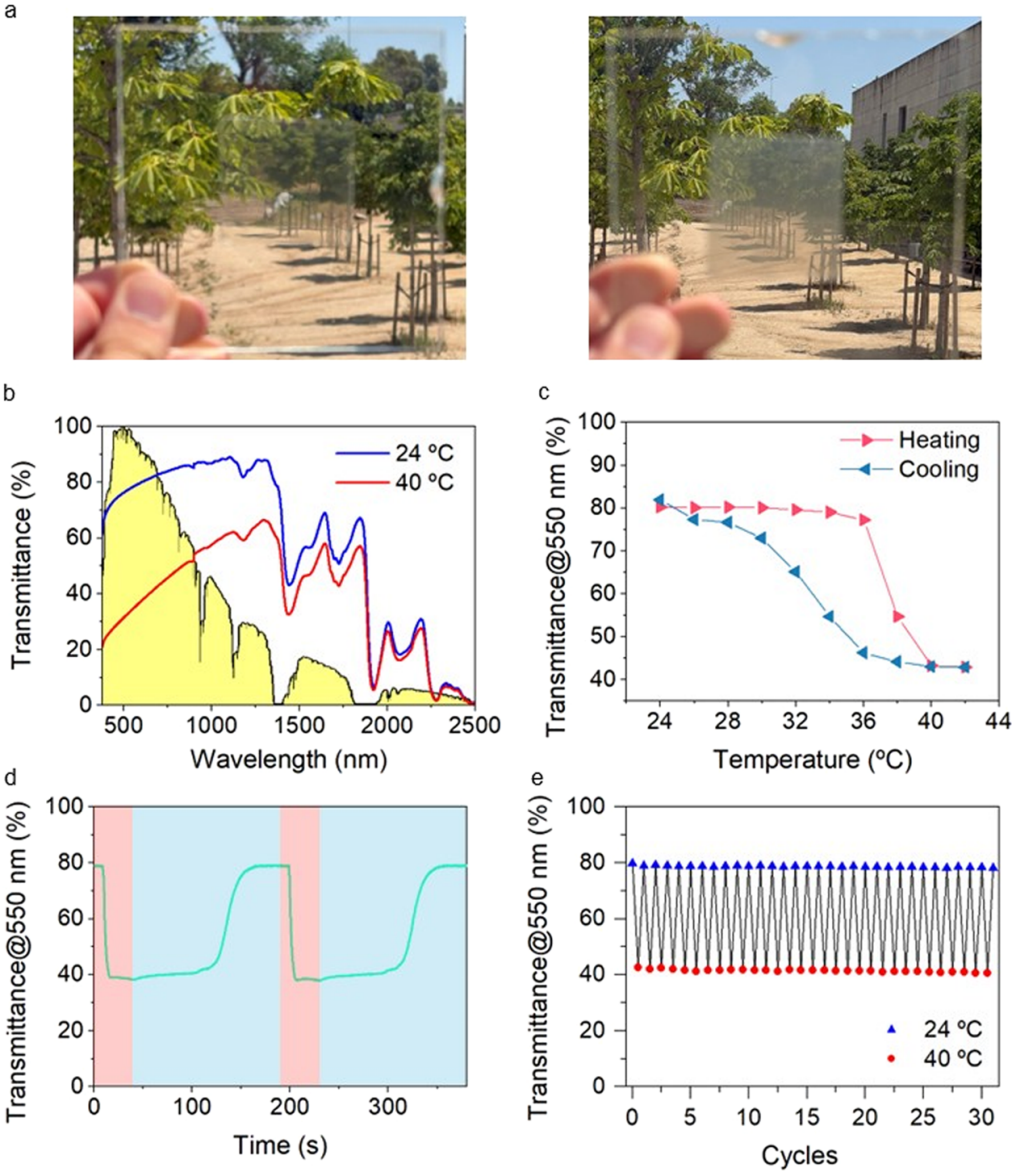
Optical properties of paraffin-acrylate composite films (0.4% wt. EC-DC particles, 19.6% wt. glycerol, 72% wt. HEMA, 8% wt. PEGDA). (a) Images of a 1.5 × 1.5 cm^2^ paraffin-acrylate film supported onto a glass substrate at room temperature and after heating above 40 °C. (b) Transmittance spectra of a paraffin-acrylate film at 24 and 40 °C. For comparison purposes, the normalized solar irradiance spectrum ASTM G173–03 Global Tilted 37° is shown superimposed. (c) Variation of transmittance at 550 nm of a paraffin-acrylate film when heating from 24 to 42 °C and subsequently cooling to recover the initial temperature. (d) Time dependent variation of transmittance at 550 nm of a paraffin-acrylate film during 2 consecutive heating–cooling cycles. Heating was conducted with a hot air flow (40–45 °C) for *t*_heating_ = 40 s (red-shaded regions), while cooling was performed at room temperature (24 °C) for *t*_cooling_ = 150 s (blue-shaded regions). (e) Variation of transmittance at 550 nm of a paraffin-acrylate film subjected to 30 consecutive heating–cooling cycles between 24 and 40 °C.

As optical attenuation due to light scattering in paraffin-acrylate composites depends exponentially on the size-dependent scattering cross-section of the particles, particle concentration and film thickness,^36^ we explored the optimization of these parameters to further improve sunlight filtering performance. First, the EC-DC particle concentration in the films was increased up to 1.6% wt., resulting in both a reduction of visible light transparency at room temperature (% Tr_550nm_^24°C^ = 68–45% for 0.6–1.6% wt. concentration) and of optical modulation upon paraffin melting (% ΔTr_550nm_^24–40°C^ = 30–20% for 0.6–1.6% wt.) (Table S3). Increasing the thickness of the films while preserving the 0.4% wt. particle concentration also proved unsuccessful, as the slightly larger transmittance modulation reached (% ΔTr_550nm_^24–40°C^ = 47% for 3 mm-thick films, Figure S4) came at the expense of substantial transparency reduction in the initial state (% Tr_550nm_^24°C^ = 59%), which is undesired for smart window coatings. Finally, we adjusted the emulsification conditions to obtain more monodisperse EC-DC particle dispersions with an average diameter of 730 nm, which is expected to provide optimal optical behavior according to Mie scattering simulations. However, similar optical properties were obtained for the resulting films bearing a 0.4% wt. particle concentration (% Tr_550nm_^25°C^ ∼ 80% and % ΔTr_550nm_^25–40°C^ ∼ 40%, Table S3), suggesting no improvement in the size homogeneity of the paraffin particle emulsions. In light of these results, future optimization of the optical performance of paraffin-acrylate films as TSWs should focus on alternative strategies to engineer paraffin particle size distribution. In spite of this, it must be noted that the optical modulation achieved with the composite films described herein matches and even exceeds the performance of some well-established TSW technologies such as liquid crystal- ^21,37^ and vanadium-oxide-based^38–42^ smart windows, which illustrates the potential of paraffin-acrylate composites to efficiently modulate sunlight transmission (Figure S5 and Table S4). In addition, these systems present other advantageous features for smart window applications, including straightforward fabrication from commercially available low-cost materials (64 € m^–2^) which produce flexible self-standing thin films that can be readily adhered to preinstalled fenestration elements (Table S4).

To confirm that the transparent-to-opaque transition exhibited by paraffin-acrylate composites originated from paraffin particle melting, additional temperature-controlled transmittance studies were conducted. When heating, the initial high transparency of our cross-linked acrylate films remained essentially constant until 36 °C (% Tr_550nm_^24–36°C^ = 80%), and it abruptly diminished upon surpassing the melting temperature of the embedded particles (% Tr_550nm_^40–42°C^ = 41%), in agreement with the loss of refractive index matching of the composite components (Figure 2c). Notably, this behavior could be fully reverted upon successive cooling (Figures 2 and S6); however, due to supercooling effects, slightly lower temperatures were required to promote paraffin particles crystallization and restore the initial refractive index matching condition with the surrounding polymer matrix, as also observed by DSC analysis of the films (Figures S3). In particular, a small thermal hysteresis width (Δ*T*_c_) of 4.5 °C was measured for the optical response of paraffin-acrylate composites, in agreement with the behavior of other paraffin-based thermochromic systems,^11^ which matches or even surpasses the performance of some of the most common TSW materials (Δ*T*_c_ = 1–5 °C, 10 °C and 10–22 °C for hydrogel-,^43–45^ copolymer-^46^ and VO_2_-based^38–42^ TSWs) (Table S4). As a result, rapid solar transmission modulation could be achieved with paraffin-acrylate films: only 13 s were required to reach 90% of opacity increment when heating with a hot air stream (40–45 °C), while 105 s were needed to recover 90% of the initial transparency upon cooling under ambient conditions (Figure 2d). Such short response times are comparable to those achieved with other fast-responsive TSW technologies (τ_heating_ = 20–40 s and τ_cooling_ = 10–80 s) for hydrogel-based TSWs,^47^ and fully meet the requirements of passive smart windows dynamically adapting to changing weather conditions. In addition, our paraffin-acrylate composites sustained multiple heating–cooling cycles without detrimentally affecting their initial transparency and the modulation of sunlight transmittance attained, thereby proving their robust TSW properties (Figure 2e).

### Resistance of Paraffin-Acrylate Composites to Weather Conditions

To validate their weathering resistance, the performance of our paraffin-acrylate composites when subjected to several relevant conditions was evaluated. First, we assessed their photo- and thermostability through an accelerated weathering test (QUV, 1000 h of strong ultraviolet radiation, 45 °C, low humidity conditions), which simulates at least 1–2 years of real sun exposure for common polymer coatings. After aging, a small decrement in transparency at room temperature was observed, with % Tr_550nm_^24°C^ diminishing from 80% to 76% (Figure 3a). However, the composite films preserved their colorless appearance and did not show the typical yellowing effect observed for many polymers during QUV tests, as no significant increment in blue light absorption was measured (Figure S7a,b).^48,49^ More importantly, the capacity of our paraffin-acrylate composites to modulate sunlight transmittance when heated was preserved, with % ΔTr_550nm_^24–40°C^ slightly rising from 39% to 45% after aging. These minor changes could not be attributed to particle migration and coalescence, nor to compositional variations induced by prolonged exposure to elevated temperatures (45 °C) and UV radiation, as evidenced by SEM, IR, and thermogravimetric analyses of the aged film (Figure S7c–f). Instead, a small increase in film thickness (4%) was observed after QUV testing, which might occur due to thermally induced reorganization of the polymer network. As experimentally determined, this effect should result in a marginal decrease in initial transparency and a slight enhancement of optical modulation, without compromising the TSW performance of paraffin–acrylate films. Therefore, these results confirm that our paraffin-based materials meet the stringent durability requirements for smart window applications.

**Figure 3:**
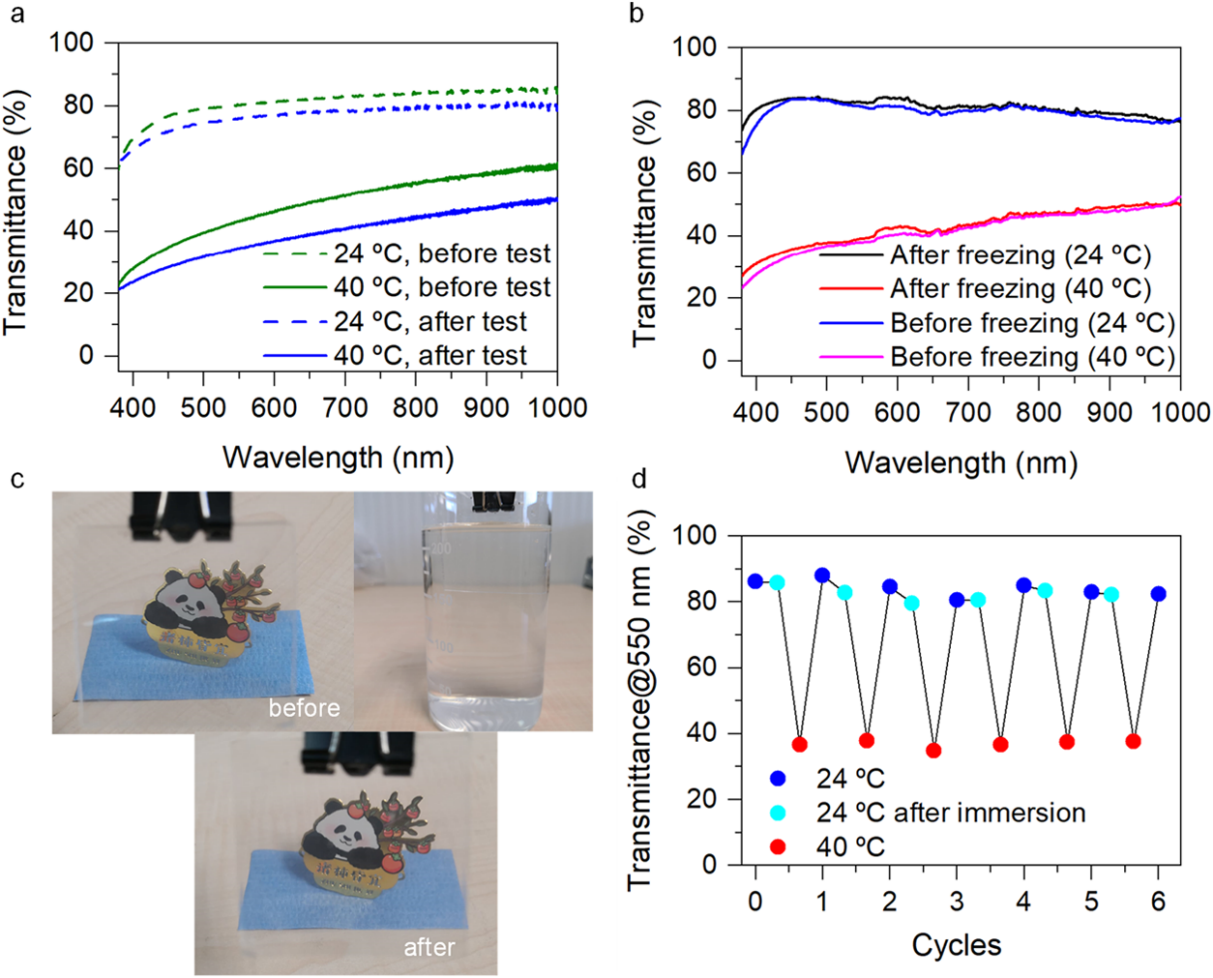
Weathering resistance of paraffin-acrylate composite films (0.4% wt. EC-DC particles, 19.6% wt. glycerol, 72% wt. HEMA, 8% wt. PEGDA). (a) Transmittance spectra of a paraffin-acrylate film at 24 and 40 °C before and after QUV testing (1000 h of strong ultraviolet radiation, 45 °C). (b) Transmittance spectra of a paraffin-acrylate film at 24 and 40 °C before and after freezing at −31 °C for 72 h. (c) Images of a 1.5 cm × 1.5 cm paraffin-acrylate film before, during and after water immersion for 5 min. (d) Variation of transmittance at 550 nm of a paraffin-acrylate film subjected to 6 consecutive cycles of water immersion for 5 min at room temperature. For each cycle, transmittance values are given for the transparent state before and after water immersion (24 °C) and for the opaque state after immersion (40 °C).

To assess the effect of low temperatures on the thermoresponsive behavior of paraffin-acrylate films, they were next subjected to freezing at −31 °C for 72 h. As shown in Figure 3b, insignificant changes were determined in transmittance before and after this thermal treatment, which did not compromise the capacity of our composite materials to modulate solar irradiation transmission. Moreover, our composites only suffered minimal changes in transmittance when the temperature was lowered to 11 °C, preserving the desired transparent state in colder regions or periods of the year (Figure S8). This is an important advantage over other TSWs based on phase change materials, which become opaque instead of remaining transparent when cooling below room temperature (e.g., liquid crystal-^31^ and ethylene glycol-based^33^ TSWs).

Finally, we investigated the water resistance of bare paraffin-acrylate films, which for PVA-based TSWs could only be achieved upon postprocess lamination with water-proof polymer layers.^11,27^ First, we evaluated the effect of high humidity levels by analyzing the optical performance of the films after 12 months of storage under ambient conditions (ca. 70% average humidity). As shown in Figure S9, marginal changes in transmittance were observed for both the transparent and opaque states of the films, indicating negligible influence of high ambient humidity. Next, we directly immersed paraffin-acrylate composites in tap water at room temperature for 5 min, and then evaluated their temperature-dependent light transmittance properties after water removal and drying. Clearly, the films preserved their integrity during this whole process, showing no signs of dissolution or degradation (Figure 3c). More importantly, negligible effects were observed on the transparency of the composites at room temperature (% Tr_550nm_^24°C^ ∼ 80%) as well as on the modulation of light transmission achieved upon heating (% ΔTr_550nm_^24–40°C^ ∼ 40%), even when they were subjected to several consecutive cycles of water immersion and drying (Figures 3d and S10a). Interestingly, a similar behavior was registered when the films were immersed for longer periods (24 h) in water under magnetic stirring, after which they showed no structural degradation effects and preserved their optical performance (Figure S10b,c). Therefore, these results substantiate the design principles explored herein for the fabrication of water-resistant, paraffin-based TSWs, making paraffin-acrylate composites suitable for outdoor applications, highly humid environments and cleaning by flushing, rinsing and scrubbing with aqueous solutions.

### Multimodal Operation of Paraffin-Acrylate Composites as Smart Windows

An attractive feature of paraffin-based TSWs is their multimodal operation, as they can be made photoresponsive by incorporating photothermal nanostructures within the composite layer, or triggered electrothermally by deposition onto transparent electrodes.^11,27^ In this work, we investigated their photothermal operation by additionally dispersing commercially available graphene oxide (GO) within the cross-linked acrylate layer. Addition of small amounts of GO preserves the neutral color desired for smart windows due to its broadband absorption within the visible region, while providing sufficient photothermal heating as to promote paraffin particle melting under strong sunlight irradiation even at low concentrations. For our paraffin-acrylate composites, a 0.01% wt. content in GO was added to minimize its effect on the initial transparency of the material in the dark, which only slightly decreased relative to GO-free samples (% Tr_550nm_^dark^ = 70%, Figure 4a). The photothermal heating generated in the resulting films was evaluated at two different ambient temperatures (23 and 27 °C) under 1 sun of irradiation (AM1.5, 100 mW cm^–2^), which simulates solar irradiance on a clear, sunny day near noon in midlatitudes. After illumination for 10 min, the surface temperature of the GO-containing composites increased ca. 11 °C in both cases due to the photothermal effects caused by GO absorption, which is about 30–35% larger than the temperature increment reached in GO-free films (Figure S11). Interestingly, the photothermal heating generated at an ambient temperature of 23 °C was not sufficient to promote paraffin melting and, consequently, efficiently block solar irradiation by inducing the transparent-to-opaque transition of the GO-doped film, as desired for moderate (and colder) temperatures. In contrast, photothermal activation of the GO-containing sample was observed at warmer ambient temperatures (27 °C), at which reduction of solar heat gain is needed. Thus, a large variation in sunlight transmission was observed at these ambient conditions under 1 sun of irradiation (% ΔTr_550nm_^dark–sunlight^ = 31%), proving that TSWs based on paraffin-acrylate layers can also be driven photothermally (Figure 4a). We also demonstrated that the photothermal operation of these materials is highly robust, as they sustained several consecutive light-induced transparent-to-opaque transitions without showing any signs of (photo)­degradation (Figure 4b). Collectively, these results prove the multiresponsive behavior of paraffin-acrylate coatings, enabling better adjustment of their TSW performance to user’s needs–i.e., switching to an opaque state only under sufficiently high temperature and irradiance.

**Figure 4:**
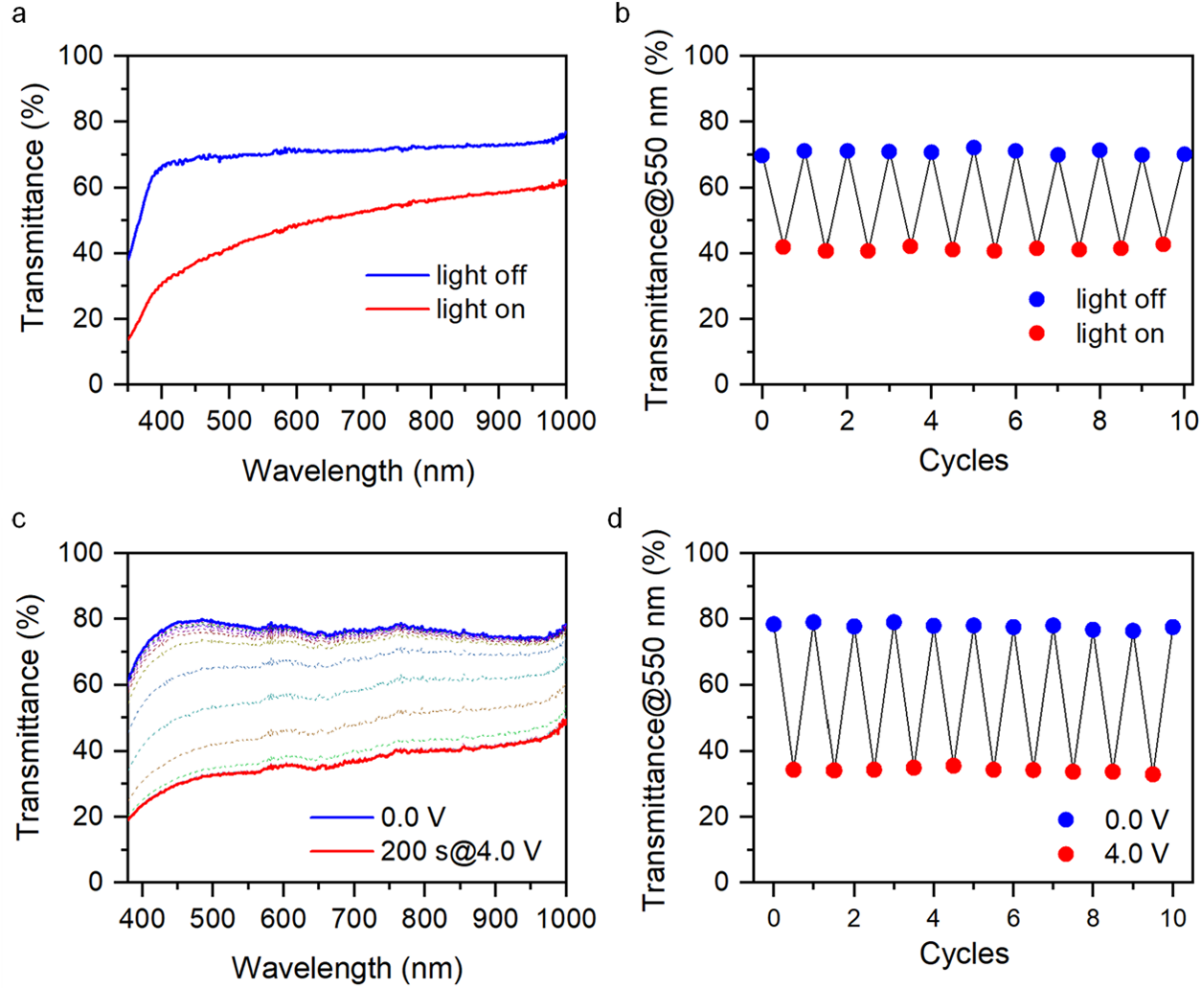
Photo- and electrothermal operation of paraffin-acrylate composite films (0.4% wt. EC-DC particles, 19.6% wt. glycerol, 72% wt. HEMA, 8% wt. PEGDA). (a) Transmittance spectra of a paraffin-acrylate film loaded with 0.01% wt. GO in the dark and under irradiation with a solar simulator (1 sun, AM1.5, 100 mW cm^–2^) for 10 min at an ambient temperature of 27 °C. (b) Variation of transmittance at 550 nm of a GO-doped paraffin-acrylate film subjected to 10 consecutive off–on cycles of solar simulator irradiation (1 sun, AM1.5, 100 mW cm^–2^) at an ambient temperature of 27 °C (*t*_off_ = 300 s, *t*_on_ = 600 s). (c) Variation of the transmittance spectra of a paraffin-acrylate film deposited onto an ITO-coated glass when applying an external voltage of 4.0 V and a current of 0.214 A. Spectra were measured every 10 s for 200 s. (d) Variation of transmittance at 550 nm of a paraffin-acrylate film deposited onto an ITO-coated glass subjected to 10 consecutive off-on cycles of electrothermal operation (*t*_off_ = 300 s, *V*_off_ = 0 V; *t*_on_ = 150 s, *V*_on_ = 4.0 V, *I*_on_ = 0.214 A).

To promote the TSW behavior of paraffin-acrylate composite films electrothermally, they were deposited onto commercially available glass substrates coated with a thin layer of indium tin oxide (ITO) as a transparent electrode (Figure S12a). The resulting device preserved the high visible light transparency of the composite in the absence of any applied voltage at room temperature (% Tr_550nm_^0V^ = 79%), with only negligible absorption contribution of ITO in this spectral window. This is a clear advantage compared to conventional electroresponsive liquid crystal-based SW technologies, where the transparent state is achieved upon voltage application and, therefore, requires energy consumption.^50,51^ When connected to an external power supply at 4.0 V, a current of 0.214 A passing through the ITO-glass electrode produced sufficient electrothermal heating to induce the transparent-to-opaque transition of the paraffin-acrylate composite within less than 150 s (% ΔTr_550nm_^0V–4V^ = 45%, Figures 4c and S12b). A similar modulation effect could be observed even at lower potentials (minimal operational voltage of 3.5 V, Figure S12c), corresponding to an estimated energy consumption of 186 mW cm^–2^. It is worth noting that these voltage values are lower than those reported for most liquid crystal-based SW systems (20–45 V)^21,36^ and other TSW materials electrothermally activated via Joule heating using transparent electrodes (6–23 V)^27,45,52,53^ (Table S4). Interestingly, this reduced voltage requirement was not achieved at the expense of response efficiency, as the time needed to reach 90% of the total optical modulation (τ = 110 s at 4.0 V) is comparable to that of other electrothermally triggered TSW systems (τ = 90–144 s).^46,47^ Electrothermal activation of the paraffin-acrylate films could be reverted by switching off the applied potential and letting the sample cool down, a process that could be repeated several times with high fatigue resistance (Figure 4d). In addition, prolonged activation by voltage application for long periods (10 h) did not cause any apparent sign of composite film degradation, as evidenced by the preservation of the original spectral response (Figure S13). Overall, these results demonstrate that the efficient modulation of solar irradiation transmittance provided by paraffin-acrylate composites can be triggered electrothermally, thereby enabling user control of this type of TSWs.

### Energy-Saving Performance

To investigate the energy-saving performance of paraffin-acrylate films as smart windows, we employed a model insulating Styrofoam box (internal dimensions: 7 cm × 7 cm × 7 cm) with a 3.0 × 2.5 cm aperture covered with a window comprising two glass panes separated by a 15 mm air gap. During energy-saving tests, this setup was irradiated with a solar simulator for 20 min at an external temperature of 28 °C, and the temperature reached by an illuminated black-colored plastic slab placed inside the model box was continuously monitored using a thermocouple (Figures 5a and S14). Four different experiments were conducted using this configuration, by coating the external face of the outer glass pane with different samples: a thermoresponsive paraffin-acrylate composite; a photothermoresponsive paraffin-acrylate composite loaded with 0.01% wt. GO; and paraffin-free, cross-linked acrylate films loaded with or without 0.01% wt. GO as controls.

**Figure 5:**
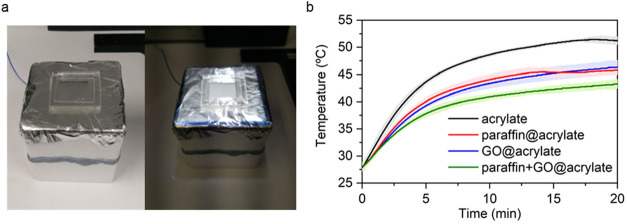
Energy-saving performance of paraffin-acrylate composite films (0.4% wt. EC-DC particles, 19.6% wt. glycerol, 72% wt. HEMA, 8% wt. PEGDA). (a) Experimental setup employed, with and without exposure to solar simulator light. (b) Variation of the temperature achieved in a black-colored plastic object (poly­(lactic acid) slab, 6 cm × 6 cm × 0.2 cm) placed in the interior of the model insulating box when irradiating with a solar simulator (1 sun, AM1.5, 100 mW cm^–2^) through a double-pane window externally coated with four different layers: a paraffin-free acrylate film (acrylate); a paraffin-free acrylate film loaded with 0.01% wt. GO (GO@acrylate); a paraffin-acrylate composite film (paraffin@acrylate); and a paraffin-acrylate composite film loaded with 0.01% wt. GO (paraffin+GO@acrylate). Irradiation was applied for 20 min at a constant external temperature of 28 °C. The shaded area around each curve corresponds to the standard deviation of two independent measurements for each acrylate coating tested.

When irradiation experiments were conducted for the double-pane window coated with a transparent, nonthermoresponsive paraffin-free acrylate film, fast solar heating gain was observed, leading to a temperature increase of Δ*T* = 23.2 °C after 20 min for the black-colored slab placed within the model insulating box (Figure 5b). This passive heating effect somewhat decreased and slowed down when an equivalent paraffin-free acrylate film was loaded with GO (Δ*T* = 18.4 °C after 20 min), whose broadband absorption causes a small reduction of sunlight transmission through the window and, therefore, of the thermal increment generated (Figure 5b). In contrast, larger reductions on solar thermal heating were observed when the double-pane window was covered with thermoresponsive and photothermoresponsive paraffin-acrylate layers. For the GO-free paraffin-acrylate-coated window, a temperature increment of Δ*T* = 17.8 °C was measured after 20 min of irradiation, which corresponds to a 23% reduction in solar heat gain relative to the paraffin-free acrylate-coated window (Figure 5b). Interestingly, a nonmonotonic temperature rise was registered for this sample, as a decrement in solar heat gain was observed after 14 min of irradiation, when the temperature of the black-colored object placed inside the model insulating box reached about 45 °C. At this point, the thermally induced transition to the opaque state of the composite film occurred, lowering sunlight transmittance and enabling a reduction of passive heating. When the window was coated with a photothermoresponsive GO-loaded paraffin-acrylate film, its transparent-to-opaque transition took place earlier at lower temperatures (5 min) due to photothermal activation. Although it did not cause an apparent variation of the solar thermal heating rate, the combined effect of increased light scattering resulting from paraffin melting and intrinsic GO light absorption led to the largest decrement in solar heat gain after 20 min of irradiation: Δ*T* = 15.2 °C, which corresponds to 34% (GO-free) and 17% (GO-loaded) reduction with respect to the nonthermoresponsive paraffin-free acrylate-coated windows. These results illustrate the potential of paraffin-acrylate films as TSWs for energy saving.

## Conclusions

In this work we have pioneered the fabrication of UV-curable thermoresponsive films as efficient smart window coatings, which are composed of eicosane-docosane microparticles, melting at ∼37 °C, that are dispersed within cross-linked acrylate matrices. When compared to other thermoresponsive smart window systems based on polymer dispersions of phase change material microstructures, our paraffin-acrylate composites show an unprecedented combination of advantageous properties. First, they are prepared from low-cost materials through fast photopolymerization from water-free formulations, avoiding the slow water-evaporation steps required in casting processes. Second, they exhibit the desired optical behavior for solar irradiation modulation, transitioning from (a) a high transparent state at low and room temperature due to refractive index matching between the solid paraffin submicrostructures and the surrounding polymer matrix (∼80% transmittance at 550 nm) to (b) a sunlight blocking state when this condition is lost by heating above paraffin melting point (∼40% transmittance at 550 nm). In addition, thanks to the structural robustness imparted by the cross-linked acrylate matrix, the materials reported herein exhibit strong water-resistance without requiring postprocessing lamination, enabling direct exposure to weather agents (e.g., rain) and treatment with aqueous solutions (e.g., for window cleaning). Finally, paraffin-acrylate films also show photothermal–by addition of graphene oxide–and electrothermal–by deposition onto transparent electrodes–responses, allowing both passive and on-demand regulation of solar irradiation as well as efficiently reducing solar heat gain indoors on sunny hot days (∼20–30%). All these features, in combination with their excellent photo- and thermal stability, substantiate the design principles explored in this work for the fabrication of paraffin-based TSWs with enhanced weathering resistance.

## Experimental Section

### Materials

All compounds were purchased from commercial providers and used as received without further purification. Eicosane >98% (EC) was acquired from TCI Chemicals. Docosane 99% (DC), glycerol >99.5% and GO (powder, 15–20 sheets, 4%–10% edge oxidized) were purchased from Merck. Poly­(vinyl alcohol) (PVA) 40–88 was purchased from Kuraray Co. 2-Hydroxylmethyl acrylate >97% (HEMA), poly­(ethylene glycol) diacrylate average *M*_n_ 700 >98% (PEGDA), 2,2-dimethoxy-2-phenylacetophenone >99% (DMPA), 1-hydroxycyclohexyl phenyl ketone >99% (HCPK) and 2-hydroxy-2-methylpropiophenone >97% (HMPP) were purchased from Sigma-Aldrich. Indium tin oxide (ITO) glass substrates (thickness: 1.1 mm; sheet resistance: ∼10 Ω sq^–1^) were purchased from Techinstro.

### Characterization Techniques

The optical transmittance of the films within the visible range was measured using an Agilent Cary 60 spectrophotometer and Agilent HP 8453 spectrophotometer in transmission mode. In this instrument, the temperature of the sample was controlled with a custom-made holder (SAPPHIRE 360) made of two joinable sapphire windows inserted in copper plates, whose temperature increased by applying an external voltage. The film of interest was placed between these two windows. To cool the film below room temperature, precooled metal clamps were applied to the holder. DSC measurements were obtained using a TAinstruments Q20. A temperature ramp rate of 5 °C min^–1^ was used for all samples. SEM images of the film surface and cross section were registered with an FEI Quanta 650 ESEM microscope applying a 5 kV voltage. The film samples were metalized with a layer of about 5 nm of Pt prior to SEM imaging. The thickness measurements from SEM images and the manual count of particle sizes in SEM images were performed using ImageJ software. IR-ATR spectra were recorded on a Bruker Tensor 27 Golden Gate spectrometer with a diamond tip. Thermogravimetric analysis was carried out with a TA Instruments TGA550 Discovery Series analyzed using a platinum HT crucible, a heating rate of 10 °C min^–1^, a heating range of 30 – 800 °C, a N_2_ flow of 40 mL min^–1^ and an air flow of 60 mL min^–1^.

The optical transmittance within the spectral range of 190–2500 nm of **ECDC@PVA** coated on glass was acquired by means of a Jasco U-770 spectrophotometer before and immediately after heating the glass substrate at about 40 °C. The transmittance modulation in the solar (300–2500 nm), luminous (390–780 nm), and NIR (780–2500 nm) ranges of the composite films was calculated as follows

1 $$T_{\mathrm{solar}/\mathrm{lum}/\mathrm{NIR}}=\frac{\int G\left(\lambda \right)T\left(\lambda \right)d\lambda }{\int G\left(\lambda \right)d\lambda }$$

2 $$\Delta T_{\mathrm{solar}/\mathrm{lum}/\mathrm{NIR}}=T_{\mathrm{solar}/\mathrm{lum}/\mathrm{NIR}}^{25{}^{\circ}C}-T_{\mathrm{solar}/\mathrm{lum}/\mathrm{NIR}}^{40{}^{\circ}C}$$

In eq 1, *T*(λ) is the wavelength-dependent measured transmittance normalized to unity, and *G*(λ) is (a) the normalized solar irradiance spectrum ASTM G173–03 Global Tilted 37° for the calculation of *T*_solar_ and *T*_NIR_, or (b) the Commission Internationale de l’Éclairage (CIE) luminous efficiency function for photopic vision for the estimation of *T*_lum_.

The accelerated aging test toward heat and UV irradiation was performed with QUV test equipment, by keeping the film for 1000 h in a chamber at 45 °C and exposed to an irradiance of 0.76 W/m^2^ (λ = 340 nm). The thermal images were acquired using FLIR ETS320 thermal imaging system.

### Preparation of Paraffin Particle Dispersions in Glycerol

360 mg of eicosane and 40 mg of docosane were melted at 80 °C in a 50 mL beaker under stirring with a cross-shaped magnetic bar. In a separate beaker, 20 g of glycerol were heated to 80 °C and then added onto the paraffin mixture under strong stirring. The mixture was let 10 min under stirring (1500 rpm) until a preemulsion was formed. The magnetic stirrer was then removed, and the preemulsion was further emulsified three times with an ultrasonic homogenizer (Branson 450 D Sonifier, 400 W to 20 kHz, equipped with disruptor horn and 13 mm flat tip) operating at 70% amplitude during 5 min in cycles of 10 s of sonication and 10 s of repose. Once the emulsion was formed, it was cooled down in an ice–water bath to induce paraffin particle crystallization and the resulting dispersion was stored in the freezer.

### Preparation of Paraffin-Acrylate Composite Films

0.080 g of PEGDA, 0.720 g of HEMA and 5 mg of DMPA were mixed and stirred until a homogeneous liquid mixture was obtained, which was then cooled to 0 °C. Next, 200 mg of a cold dispersion of EC-DC particles in glycerol were added. The mixture obtained was manually mixed and promptly introduced into a square-shaped, 1.5 cm × 1.5 cm mold made of two parallel glass substrates separated by double-sided tape (thickness ∼ 1.0 mm, Figure S1a), which enabled good uniformity and reproducibility of film thickness (standard deviation <0.04 mm). To promote acrylate polymerization and cross-linking, the liquid formulation was irradiated for 60–120 s at 365 nm in the interior of a UV photoreactor (CL-3000L UVP Cross-linker). The sample was then left to cure for 8 h in the dark, after which the two glass substrates were separated and the self-standing polymer layer produced was detached from the mold. Finally, it was left to dry overnight for better transparency. A similar procedure was followed for the preparation of other acrylate films in this work, although with slight differences. For GO containing films, 10 mg of commercially available graphene oxide were added to 10 g of HEMA and they were dispersed with an ultrasonic homogenizer (Branson 450 D Sonifier, 400 W to 20 kHz, equipped with disruptor horn and 13 mm flat tip) operating at 70% amplitude during 5 min in cycles of 10 s of sonication and 10 s of repose. The mixture was diluted 7 times with HEMA before adding the EC-DC particle suspension, PEGDA and DMPA. For the preparation of paraffin-free acrylate layers, 200 mg of glycerol were added to the PEGDA-HEMA-DMPA mixture.

## Supplementary Material



## Data Availability

Raw data supporting the findings presented in the main text and the Supporting Information are available on https://dataverse.csuc.cat/.

## Notes

Additional data on the preparation and characterization of thermoresponsive smart window films based on paraffin-polymer composites (PDF)

## Abbreviations

DMPA: 2,2-dimethoxy-2-phenylacetophenone
DC: docosane
DSC: differential scanning calorimetry
EC: eicosane
GO: graphene oxide
HCPK: 1-hydroxycyclohexyl phenyl ketone
HEMA: 2-hydroxyethylmethacrylate
HMPP: 2-hydroxy-2-methylpropiophenone
HPMC: hydroxypropyl methylcellulose
ITO: indium tin oxide
PEGDA: poly­(ethyleneglycol) dimethacrylate
PVA: poly­(vinyl alcohol)
QUV: accelerated aging test
RI: refractive index
SEM: scanning electron microscopy
SW: smart window
TGA: themogravimetric analysis
TSW: themoresponsive smart window.
