# Supplementary material for: Weather-Resistant Thermoresponsive UV-Curable Smart Window Composites Based on Paraffin Particles
Source: ACS Appl Mater Interfaces. 2026 Jun 25;18(26):37058–68. doi: 10.1021/acsami.6c04877 (PMC13352511; doi:10.1021/acsami.6c04877)
Supplement: Supplementary file 1 [file am6c04877_si_001.pdf]

Supporting Information for:

Weather-Resistant Thermoresponsive UV-Curable Smart Window Composites Based on Paraffin Particles

Marc Villabona,[1] Lorenzo Vallan,[2] Daniel Ruiz-Molina,[2] Claudio Roscini,[2,] Jordi Hernando[1,]**

[1] Departament de Química, Universitat Autònoma de Barcelona, Edifici C/n, Campus UAB, 08193 Cerdanyola del Vallès, Spain. E-mail: jordi.hernando@uab.cat

[2] Catalan Institute of Nanoscience and Nanotechnology (ICN2), CSIC and BIST, Campus UAB, Bellaterra, 08193 Barcelona, Spain. E-mail: claudio.roscini@icn2.cat

Content

Supporting Tables……………………………………………..…..……….S2

Supporting Figures……………………………………..…………….…S8

Estimation of scattering cross-sections and coefficients…………………S17

References………………….…………………………………………S19

Supporting Tables

Table S1. Experimental conditions used to optimize the preparation of non-aqueous dispersions of EC-DC particles.[a]

Liquid phase	Amount of EC-DC[b] (% wt.)	Emulsification conditions[c]	Result
HEMA	1	N/A	Miscible
PEGDA	1	N/A	Miscible
PEGDA:Glycerol 1:1	N/A	N/A	Not Miscible
Glycerol	2%	5 min, 70%, 10 s/10 s	Emulsion
**Glycerol**	**2%**	**10 min, 70%, 10 s/10 s**	**Emulsion**
Glycerol	2%	15 min, 70%, 10 s/10 s	Emulsion[d]
Glycerol	2%	20 min, 70%, 10 s/10 s	Emulsion[d]
Glycerol	2%	25 min, 70%, 10 s/10 s	Emulsion[d]
Glycerol	3%	10 min, 70%, 10 s/10 s	Emulsion[e]
Glycerol	4%	10 min, 70%, 10 s/10 s	Emulsion[e]
Glycerol	8%	10 min, 70%, 10 s/10 s	Emulsion[e]

[a] Best conditions are shown in bold. [b] 9:1 mass ratio between EC-DC. [c] Total emulsification time, sonication amplitude, on-off cycling times for each sonication period. [d] No benefit was observed upon increasing the emulsification time above 10 min (see Table S3). [e] The films made from these emulsions containing higher amounts of EC-DC particles were opaque (see Table S3).

Table S2. Experimental conditions used to optimize the photopolymerization of liquid dispersions of EC-DC particles in mixtures of glycerol, HEMA and PEGDA.[a]

**HEMA** (% wt.)	**PEGDA** (% wt.)	**Glycerol dispersion**[b] (% wt.)	**Initiator**[c]	**Irradiation time**[d] (s)	**Result**
90	10	0	DMPA (1% wt.)	120	Solid Polymer
90	10	0	DMPA (0.5% wt.)	120	Solid Polymer
90	10	0	DMPA (0.2% wt.)	120	No solid Polymer
90	10	0	DMPA (0.5 % wt.)	60	Solid Polymer
90	10	0	DMPA (0.5% wt.)	30	No solid Polymer
90	10	0	HCPK (0.5% wt.)	120	Solid Polymer
90	10	0	HCPK (0.5% wt.)	60	No solid Polymer
90	10	0	HMPP (0.5% wt.)	120	Solid Polymer
90	10	0	HMPP (0.5% wt.)	60	No solid Polymer
85.5	4.5	5	DMPA (0.5 % wt.)	60	Solid Polymer
81	9	10	DMPA (0.5 % wt.)	60	Solid Polymer
**72**	**8**	**20**	**DMPA (0.5 % wt.)**	**60**	**Solid Polymer**
67.5	7.5	25	DMPA (0.5 % wt.)	60	Solid Polymer
76	4	20	DMPA	60	Soft Polymer

			(0.5 % wt.)		
64	16	20	DMPA (0.5 % wt.)	60	Brittle Polymer
48	32	20	DMPA (0.5 % wt.)	60	Brittle Polymer

[a] Best conditions are shown in bold. [b] Dispersion of 9:1 EC-DC particles (1.5% wt.) in glycerol. [c] DMPA: 2,2-dimethoxy-2-phenylacetophenone; HCPK: 1-hydroxycyclohexyl phenyl ketone; HMPP: 2-hydroxy-2-methylpropiophenone. [d] At 365 nm in a UV photoreactor (247 mJ cm^{-2} min^{-1}).

Table S3. Experimental conditions for the optimization of the composition of paraffin-acrylate films to maximize their thermoresponsive optical behavior.[a]

**HEMA** (% wt.)	**PEGDA** (% wt.)	**Glycerol dispersion** (% wt.)	**EC-DC in the dispersion** (% wt.)	**Emulsification conditions**	**Result**[b,c]
72	8	20	1.5	10 min, 70%,<br>10 s/10 s	% $Tr^{24\,°C}_{550\,nm}$ = 78%,<br>$\Delta Tr^{24\text{-}40\,°C}_{550\,nm}$ = 30%
**72**	**8**	**20**	**2**	**10 min, 70%,<br>10 s/10 s**	**% $Tr^{24\,°C}_{550\,nm}$ = 79%,<br>$\Delta Tr^{24\text{-}40\,°C}_{550\,nm}$ = 40%**
72	8	20	3	10 min, 70%,<br>10 s/10 s	% $Tr^{24\,°C}_{550\,nm}$ = 68%,<br>$\Delta Tr^{24\text{-}40\,°C}_{550\,nm}$ = 30%
72	8	20	4	10 min, 70%,<br>10 s/10 s	% $Tr^{24\,°C}_{550\,nm}$ = 55%,<br>$\Delta Tr^{24\text{-}40\,°C}_{550\,nm}$ = 25%
72	8	20	8	10 min, 70%,<br>10 s/10 s	% $Tr^{24\,°C}_{550\,nm}$ = 45%,<br>$\Delta Tr^{24\text{-}40\,°C}_{550\,nm}$ = 20%
85.5	4.5	5	2	10 min, 70%,<br>10 s/10 s	% $Tr^{24\,°C}_{550\,nm}$ = 80%,<br>$\Delta Tr^{24\text{-}40\,°C}_{550\,nm}$ = 2%
81	9	10	2	10 min, 70%,<br>10 s/10 s	% $Tr^{24\,°C}_{550\,nm}$ = 79%,<br>$\Delta Tr^{24\text{-}40\,°C}_{550\,nm}$ = 5%
67.5	7.5	25	2	10 min, 70%,<br>10 s/10 s	% $Tr^{24\,°C}_{550\,nm}$ = 20%,<br>$\Delta Tr^{24\text{-}40\,°C}_{550\,nm}$ = 5%
72	8	20	2	5 min, 70%,<br>10 s/10 s	% $Tr^{24\,°C}_{550\,nm}$ = 80%,<br>$\Delta Tr^{24\text{-}40\,°C}_{550\,nm}$ = 35%
72	8	20	2	15 min, 70%,<br>10 s/10 s	% $Tr^{24\,°C}_{550\,nm}$ = 79%,<br>$\Delta Tr^{24\text{-}40\,°C}_{550\,nm}$ = 39%
72	8	20	2	20 min, 70%,<br>10 s/1 0s	% $Tr^{24\,°C}_{550\,nm}$ = 80%,<br>$\Delta Tr^{24\text{-}40\,°C}_{550\,nm}$ = 40%
72	8	20	2	25 min, 70%,<br>10 s/10 s	% $Tr^{24\,°C}_{550\,nm}$ = 79%,<br>$\Delta Tr^{24\text{-}40\,°C}_{550\,nm}$ = 38%

[a] Best conditions are shown in bold. [b] Irradiation at 365 nm in a UV photoreactor for 90 s (247 mJ cm^{-2} min^{-1}) using DMPA as a photoinitiator (0.5% wt.). [c] Emulsion kept in the freezer for 7 days before use.

Table S4. Comparison of the performance of paraffin-acrylate smart window coatings with other TSW materials.

Ref	Material	% $Tr_{\mathrm{lum}}^{\mathrm{rt}}$ [a]	color^{trans} [b]	%ΔTr_{solar} [c]	T_c (ΔT_c) (°C) [d]	Self-standing film [e]	Water resistance [f]	Operation modes [h]	Voltage (V) [i]	Cost (€ m^{-2}) [k]
**This work**	**paraffin-acrylate**	**78.0**	**Colorless**	**37.8**	**37 (4.5)**	**Yes**	**Yes**	**T, P, E**	**3.5-4.0**	**64**
[1]	paraffin-PVA	77.3	Colorless	58.6	37 (4.5)	Yes	No	T, P	-	26
[2]	paraffin-PVA	71.0	Colorless	41.6	37 (4.5)	Yes	No	T, P, E	6.0	54
[3]	hydrogel	87.2	Colorless	81.3	32 (1.0)	No (liquid suspension)	-	T	-	293
[4]	hydrogel	87.0	Colorless	70	32 (5.0)	No (liquid suspension)	-	T	-	1360
[5]	hydrogel	89.1	Colorless	69	32	No (liquid suspension)	-	T	-	106
[6]	hydrogel	99.0	Colorless	58.2	32 (4.0)	No (liquid suspension)	-	T, P	-	-
[7]	liquid crystal	57.8	Slightly yellow	34.6	31.0 (1.0)	Yes	- [g]	T, E	35	-
[8]	liquid crystal	55.0	Slightly yellow	40.9	42.5	Yes	- [g]	T, E	20 or 45 [j]	-
[9]	copolymer	80.9	Colorless	62.7	30 (10)	Yes	- [g]	T, E	6	95
[10]	VO_2	41.5	Colored	3.1	68	No	-	T	-	-
[11]	VO_2	52.2	Colored	17.3	40 (22.3)	No	-	T	-	-

[12]	VO_2	32.4	Colored	8.6	61.5 (15)	No	-	T	-	-
[13]	VO_2	55.9	Colored	6.9	64.3	No	-	T	-	-
[14]	VO_2	73.4	Colored	18.2	42 (20)	No	-	T	-	-
[15]	VO_2	62.7	Colored	20.8	68	No	-	T	-	-
[16]	VO_2	61.7	Colored	11.7	43 (16.9)	No	-	T	-	-
[17]	VO_2	29.6	Colored	15.4	68 (10)	No	-	T, E	24	-
[18]	VO_2	57.3	Colored	13.8	50 (18.6)	No	-	T, E	13	-

[a] Visible light transmittance in the transparent state at room temperature. [b] Color of the transparent state. [c] Transmittance modulation over the solar irradiance spectrum between the transparent and opaque states. [d] Temperature (T_c) for the transparent-to-opaque transition upon heating and thermal hysteresis width (ΔT_c), if reported. [e] Self-standing films do not require a glass substrate (or a glass container). [f] Only for self-standing films. [g] No data provided about water resistance. [h] Operation modes: T = thermochromic; P = photothermochromic; E = electrothermochromic or electrochromic. [i] Voltage required for maximal solar transmittance modulation in the electrothermochromic/ electrochromic mode. [j] 20 V for electrothermochromic operation and 45 V for electrochromic operation. [k] Cost was only estimated for those films which did not require any synthesis step, using the price for the different components of the materials obtained from chemical distributors. The price of the substrate was not considered.

Supporting Figures

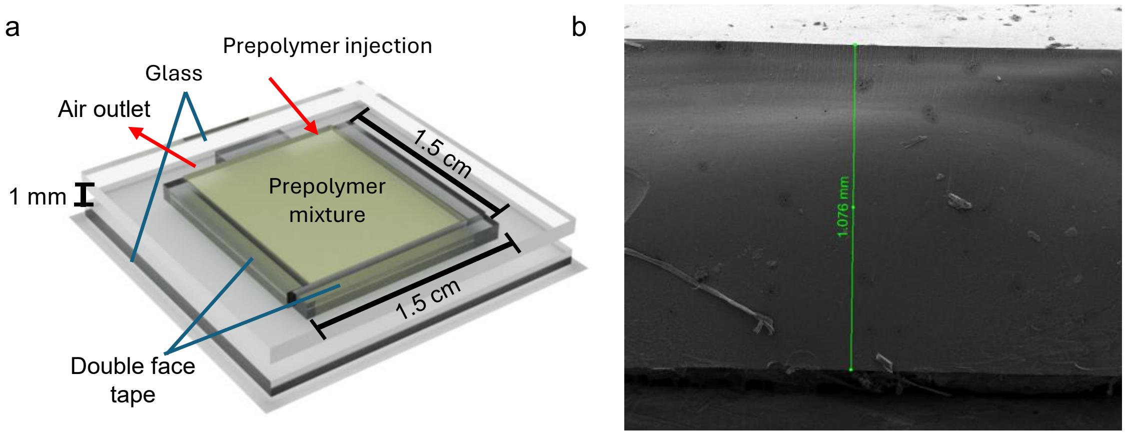

Figure S1. (a) Experimental scheme used for the preparation of paraffin-acrylate composite films by photopolymerization of liquid dispersions of EC-DC particles in a mixture of HEMA, PEGDA, glycerol and DMPA. (b) SEM image of an EC-DC@HEMA-PEGDA film cross section.

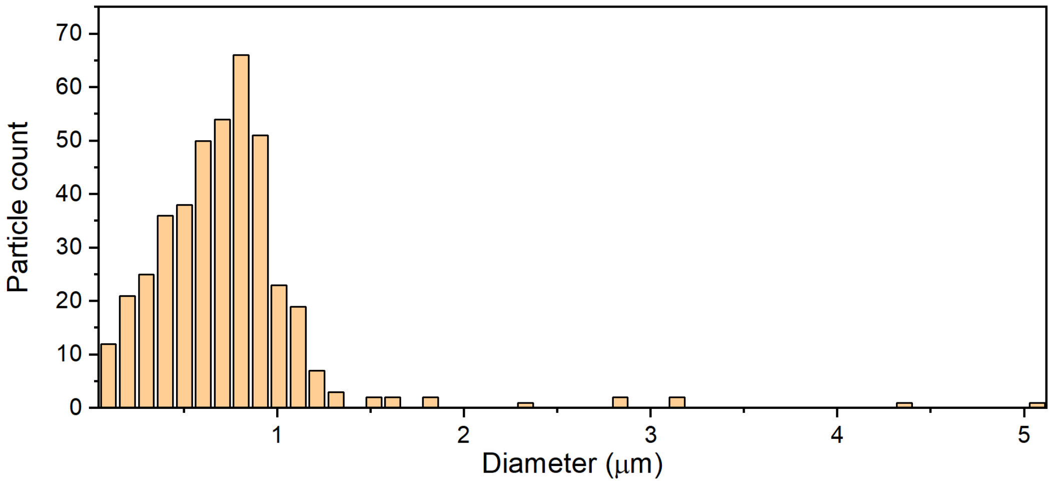

Figure S2. Diameter distribution obtained by SEM imaging of the voids generated on the edge surface of paraffin-acrylate composites after treatment with hexane to selectively dissolve EC-DC particles (average diameter = 730 nm).

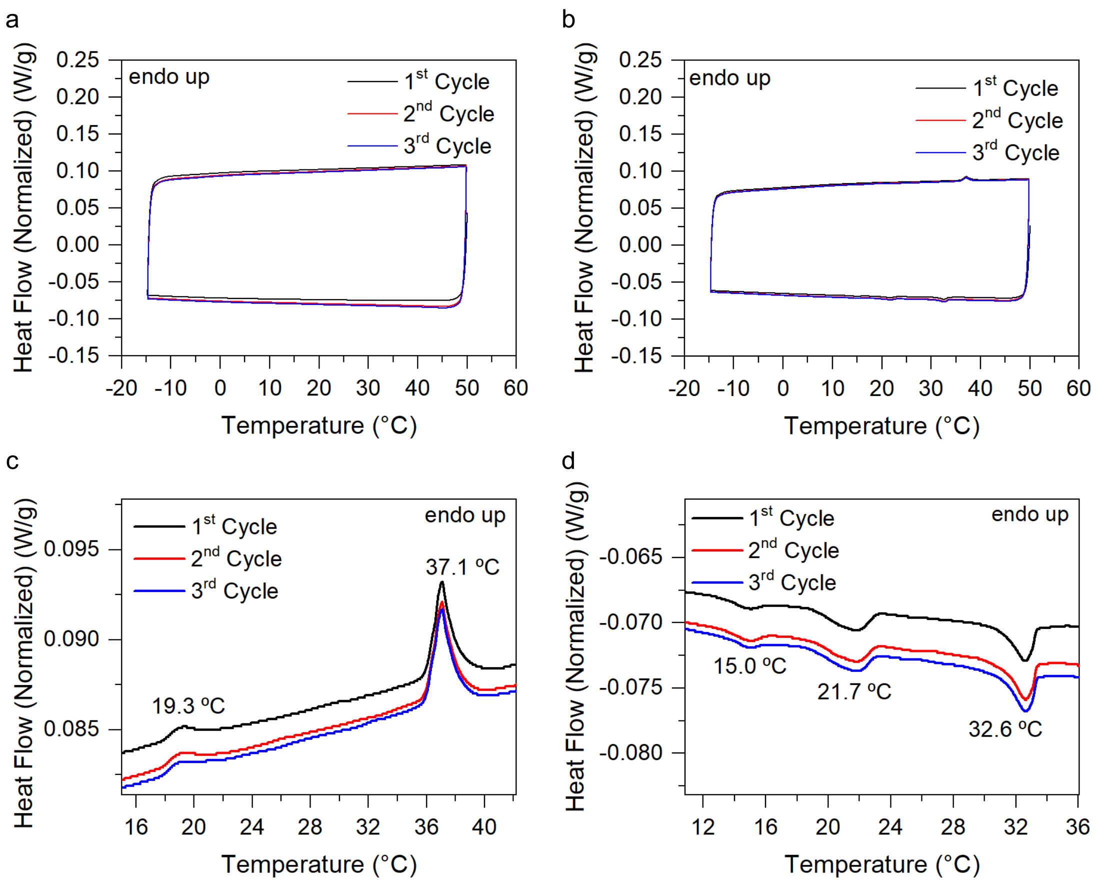

Figure S3. DSC thermograms of (a) a paraffin-free cross-linked acrylate film, and (b) a paraffin-acrylate composite film, which were measured during 3 consecutive heating-cooling cycles. While no phase transitions were observed for the paraffin-free sample, several endothermic and exothermic peaks were measured for the composite layer that are indicative of paraffin particle presence. Amplified views of these peaks for the paraffin-acrylate composite film are shown in (c) and (d). Two peaks were observed when heating, which are characteristic of solid-solid ($T \sim 19$ ºC) and solid-liquid ($T \sim 37$ ºC) phase transitions for EC-DC 9:1 particles. An additional peak was measured at $T \sim 22$ ºC when cooling that could be attributed to the formation of an additional metastable solid phase, as often observed in paraffin particles.[19]

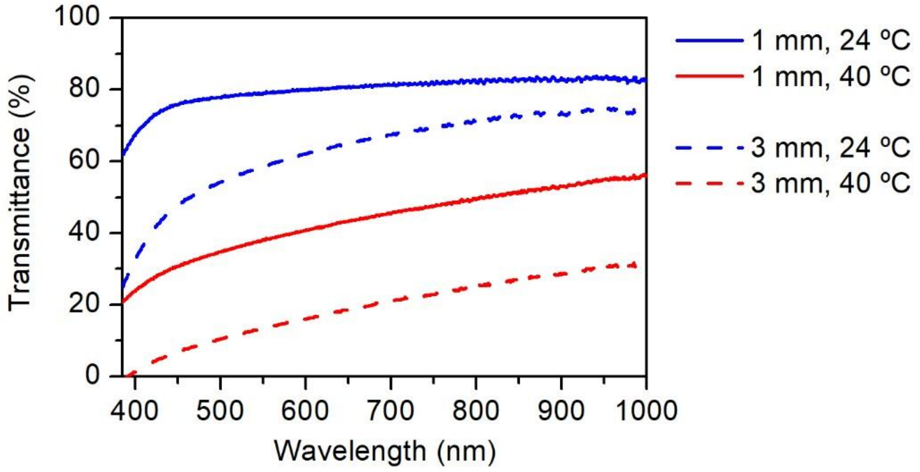

Figure S4. Transmittance spectra of paraffin-acrylate films of equivalent paraffin particle concentration but different thickness (1 mm and 3 mm) in their initial state at room temperature (24 ºC) and after heating to 40 ºC to induce the transparent-to-opaque transition. Light transparency at 550 nm and 24 ºC was measured to be % $Tr^{24\,^{\circ}\mathrm{C}}_{550\ \mathrm{nm}}$ = 79% and 59% for the 1 mm- and 3 mm-thick films, respectively, while light transmittance modulation achieved at 550 nm upon heating was estimated to be % $\Delta Tr^{24\text{-}40\,^{\circ}\mathrm{C}}_{\mathrm{lum}}$ = 40% and 47%, respectively.

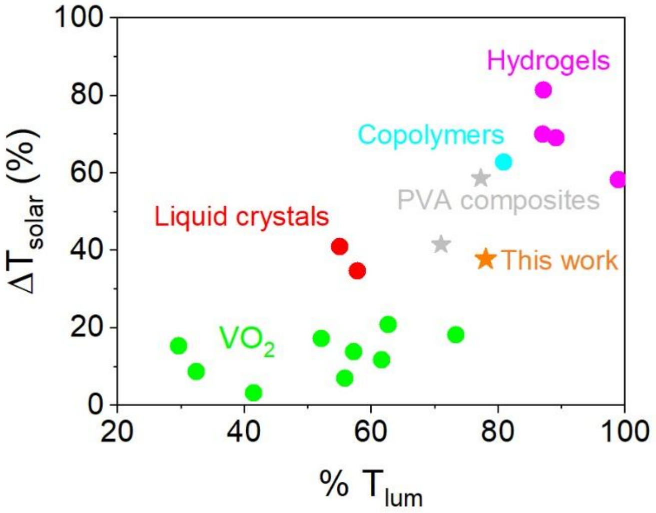

Figure S5. Comparison of the thermal variation of solar transmittance between the transparent and opaque states (% $\Delta Tr_{\mathrm{solar}}$) and of the luminous transmittance in the transparent state (% Tr_{lum}) of our paraffin-acrylate composite films relative to previously reported thermoresponsive smart windows based on paraffin-PVA composites,[1,2] hydrogels,[3-6] liquid crystals,[7,8] phase change copolymers,[9] and vanadium oxide.[10-18]

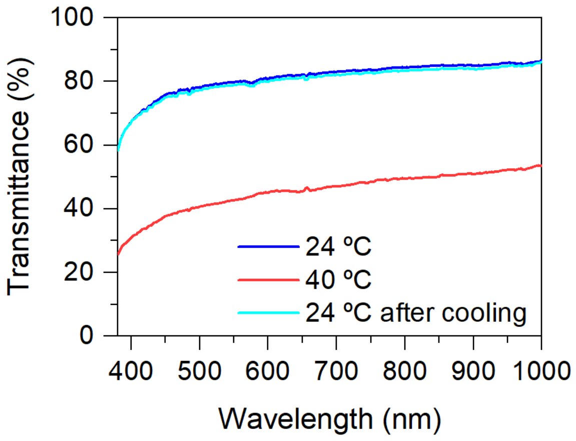

Figure S6. Transmittance spectra of a paraffin-acrylate film initially at room temperature (24 ºC), after heating to 40 ºC to induce the transparent-to-opaque transition, and after letting it cool down to room temperature again (24 ºC after cooling).

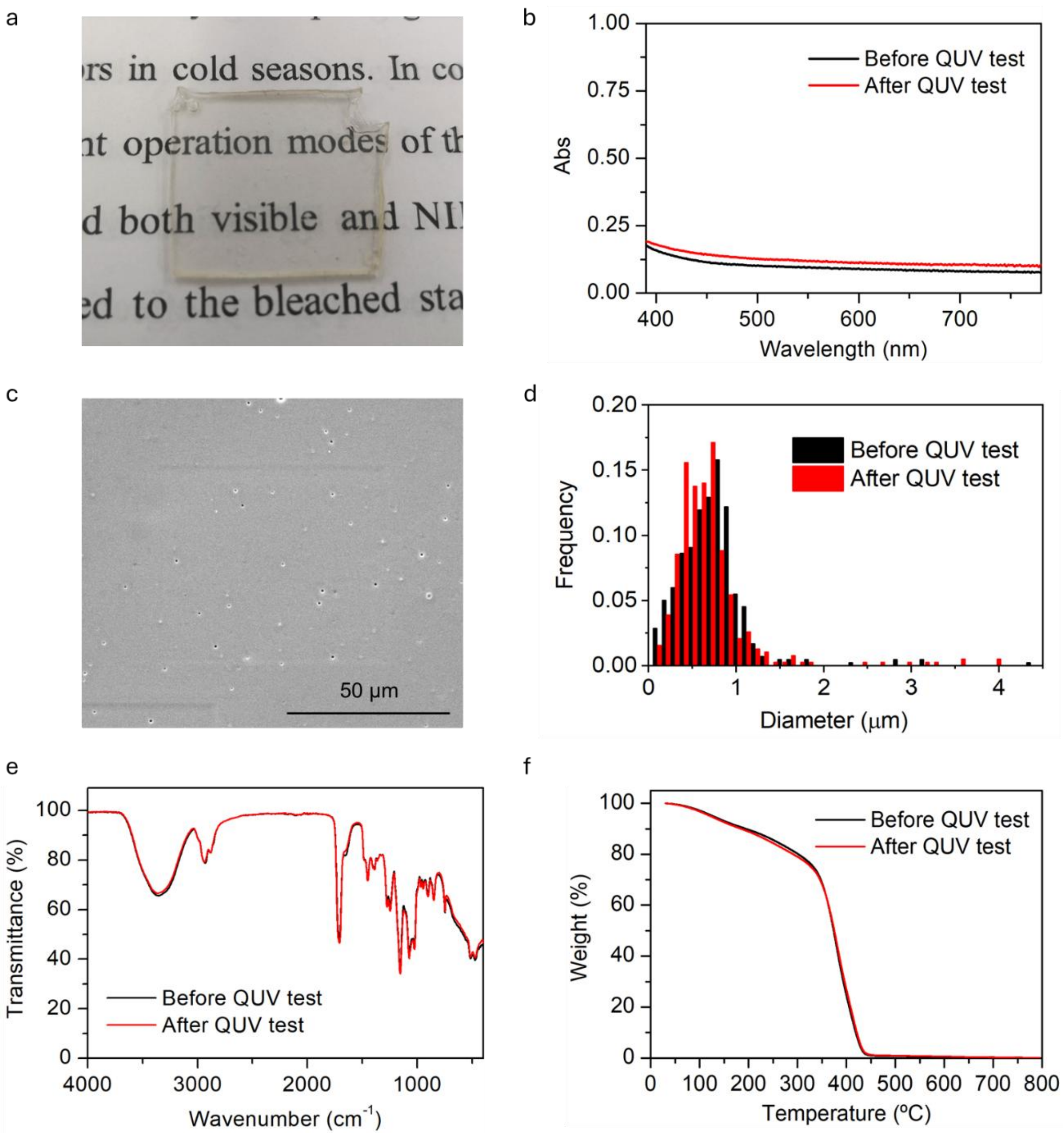

Figure S7. (a) Photograph of a paraffin-acrylate film after being subjected a QUV test (1000 h of strong ultraviolet radiation, 45 °C). (b) Absorption spectra in the visible region of the transparent state (24 ºC) of the film before and after being subjected to the QUV test. No yellowing of the film was observed after the QUV ageing test, which would otherwise result in a selective increase in absorption in the violet-blue region (ca. 390-500 nm). (c) SEM image of the cross-section of a paraffin-acrylate composite film after QUV ageing test, which was previously treated with hexane to dissolve the EC-DC particles exposed at the surface. (d) Diameter distribution obtained by SEM imaging of the voids generated on the edge surface of paraffin-acrylate composites after treatment with hexane to selectively dissolve EC-DC particles. Data is shown for a freshly prepared film (average diameter = 730 nm) and a film subjected to a QUV test (average diameter

= 700 nm). (e) ATR-IR spectra and (f) TGA thermograms of a freshly prepared paraffin-acrylate film and of a film subjected to a QUV test.

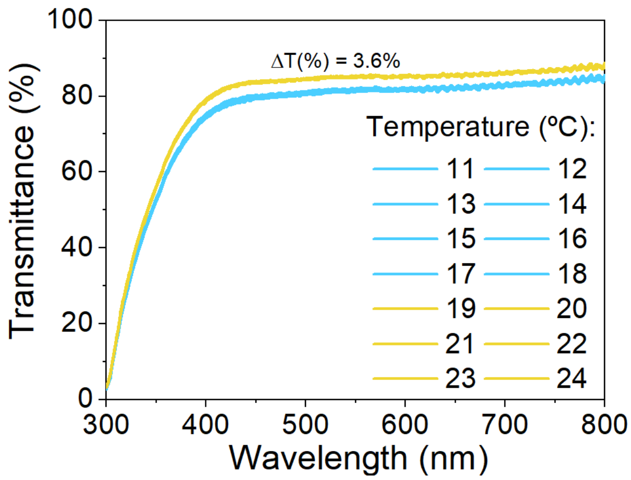

Figure S8. Transmittance spectra of the composite film while kept below room temperature. The transmittance suffered only a slight change which did not affect the TSW composite transparency.

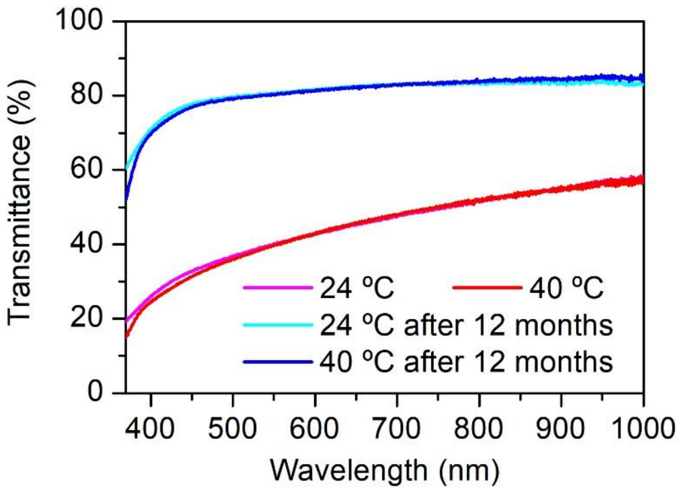

Figure S9. Transmittance spectra of the transparent (24 ºC) and opaque (40 ºC) states of paraffin-acrylate films freshly prepared and after storage for 12 months under high humidity ambient conditions (ca. 70% annual average relative humidity in the Barcelona area).

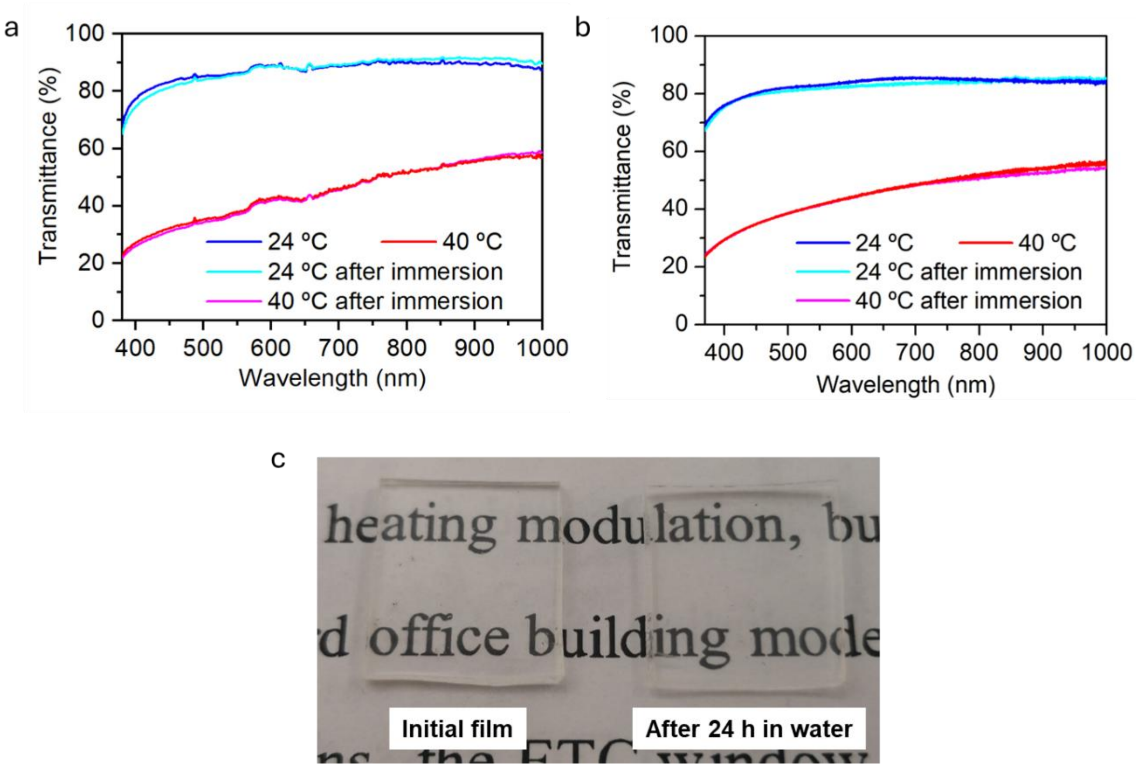

Figure S10. (a-b) Transmittance spectra of the transparent (24 ºC) and opaque (40 ºC) states of a paraffin-acrylate film before and after immersion in water for (a) 5 min and (b) 24 h under constant magnetic stirring. (c) Photographs of paraffin-acrylate films before and after immersion in water for 24 h under magnetic stirring.

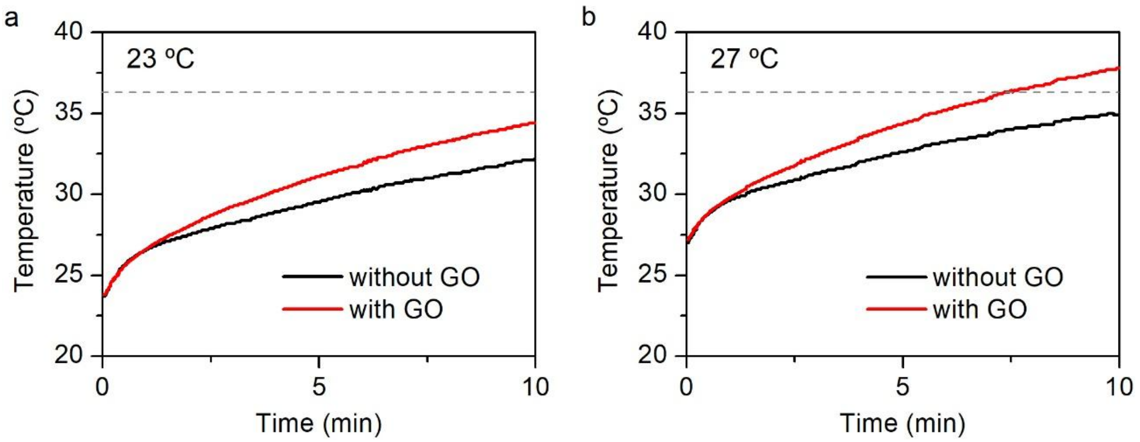

Figure S11. Variation of the temperature on the surface of paraffin-acrylate films when irradiated with a solar simulator (1 sun, AM1.5, 100 mW cm^{-2}) for 10 minutes at two different ambient temperatures: (a) 23 ºC, and (b) 27 ºC. Results are shown for films with and without doping with 0.01% wt. GO. The dashed line represents the melting temperature of the paraffin particles of the films, over which they become opaque.

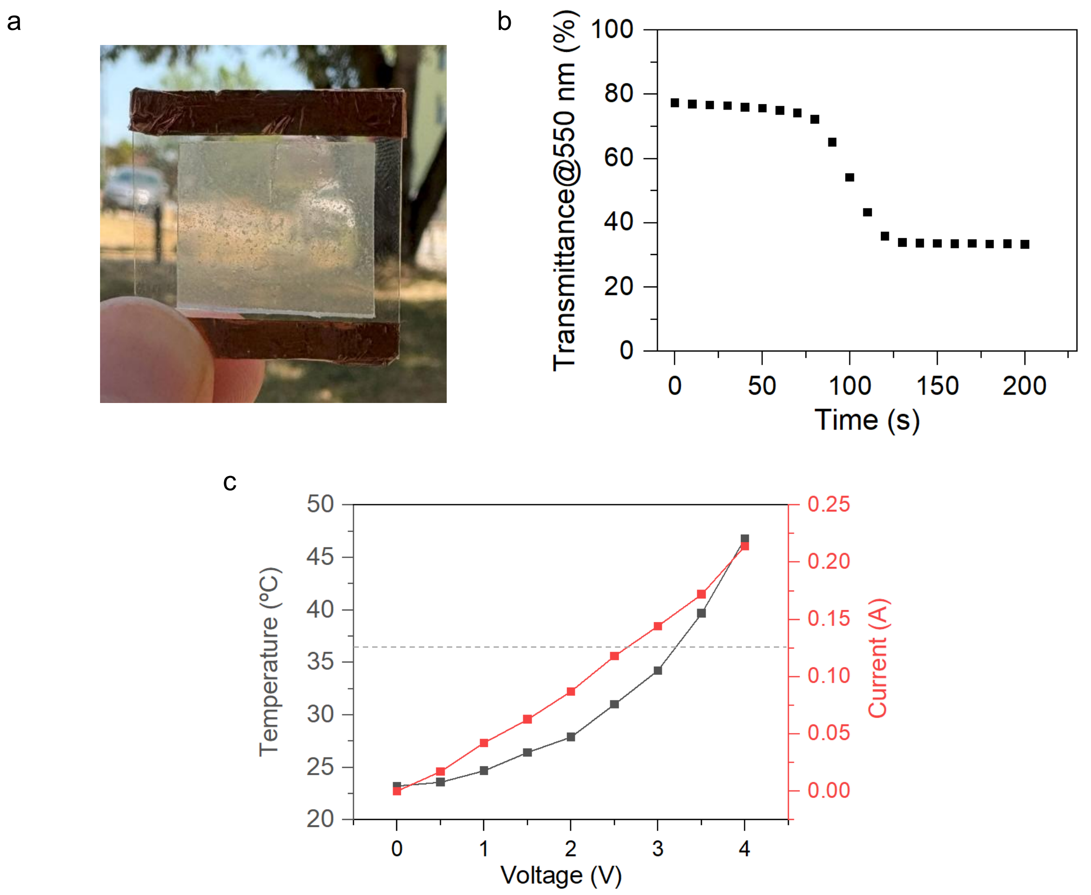

Figure S12. (a) Image a 1.5 x 1.5 cm paraffin-acrylate film deposited onto an ITO-coated glass substrate, which was covered with copper tape in its upper and lower edges to facilitate power supply connection. The opaque state of the system is shown after application an external voltage of 4.0 V and passing a current of 0.214 A for 150 s. (b) Variation of transmittance at 550 nm of the paraffin-acrylate film showed in a) during the application of the external electrical current. After 120 s, sufficient heat was electrothermally generated by Joule effect in the ITO electrode as to promote complete transparent-to-opaque transition of the composite film. (c) Temperature reached by the film upon increasing voltage and current. The dashed line represents the melting temperature of the paraffin particles of the films, over which they become opaque.

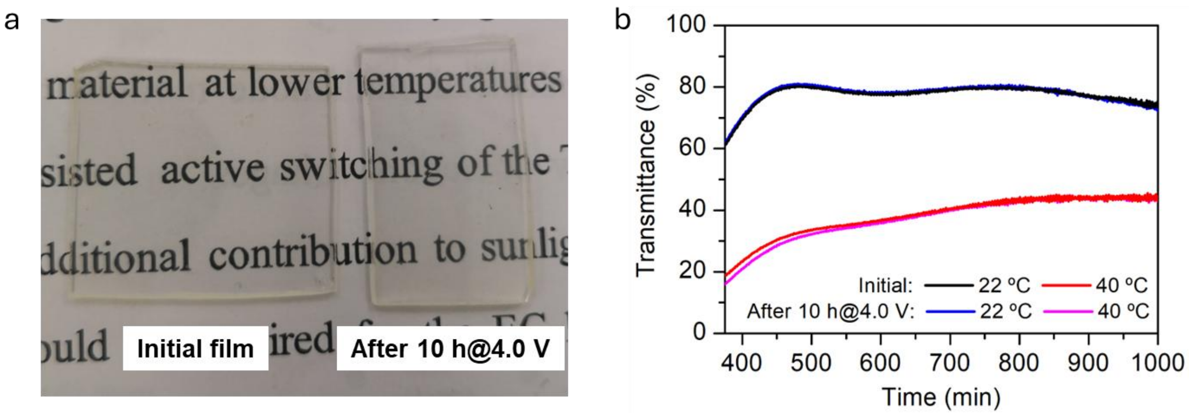

Figure S13. (a) Images a freshly made paraffin-acrylate film and of a paraffin-acrylate film subjected to an external voltage of 4.0 V during 10 h (I = 0.214 A) after deposition onto an ITO-coated glass substrate. (b) Transmittance spectra of the transparent (22 ºC) and opaque (40 ºC) states of a paraffin-acrylate film before and after being subjected to an external voltage of 4.0 V during 10 h (I = 0.214 A) after deposition onto an ITO-coated glass substrate.

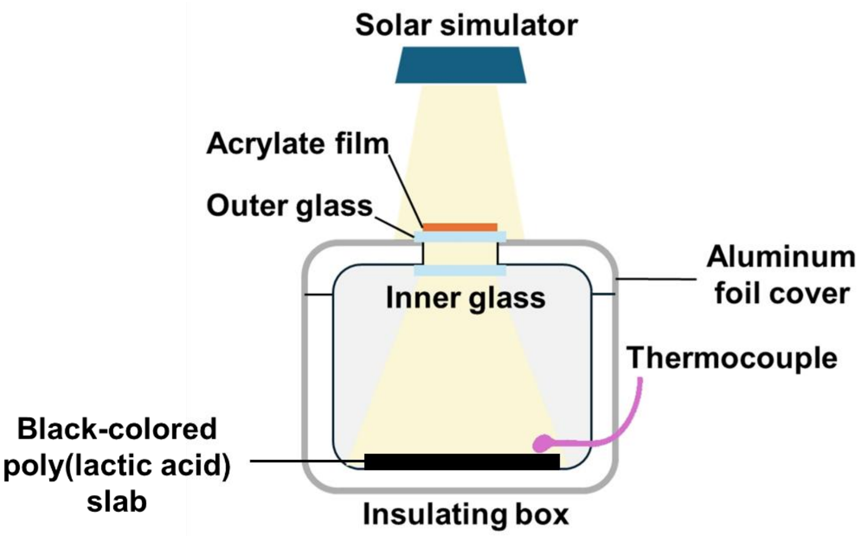

Figure S14. Image of the experimental setup used for the energy-saving experiments.

Estimation of scattering cross-sections and light attenuation coefficients

1) Calculation of scattering cross-sections

For the calculation of $\sigma_{scattering}$, we used an open-source code for Mie scattering simulations of single nano- and microspheres in a dielectric medium.[20] The following input values were used in the simulations:

- EC-DC particle diameter: 730 nm
- Refractive index of EC-DC particles at 25 ºC: 1.5144 (obtained from bulk EC at 568 nm, see Figure 1b)
- Refractive index of EC-DC particles at 40 ºC: 1.4344 (obtained from bulk EC at 568 nm, see Figure 1b)
- Refractive index of acrylate film at 25 ºC: 1.5064 (obtained from a paraffin-free acrylate film with the same composition as the paraffin-acrylate composite films, see Figure 1b)
- Refractive index of acrylate film at 40 ºC: 1.5016 (obtained from a paraffin-free acrylate film with the same composition as the paraffin-acrylate composite films, see Figure 1b)

The $\sigma_{scattering}$ values obtained at 550 nm were:

- $\sigma_{scattering}^{550\text{ nm}}$ at 25 ºC = 876.8 nm^2
- $\sigma_{scattering}^{550\text{ nm}}$ at 40 ºC = $5.740 \cdot 10^4$ nm^2

2) Calculation of visible light attenuation coefficients

First, the concentration of EC-DC particles in the paraffin-acrylate films was estimated:

- Film volume: 1.5 x 1.5 x 0.1 $cm^3 \Rightarrow v_{film} = 0.225\ cm^3$
- Mass per particle: $\rho_{EC} \approx \rho_{DC} = 0.778\ g\ cm^{-3}$, $d_{particle} = 730\ nm \Rightarrow m_{particle} = 1.585 \cdot 10^{-13}\ g$
- Number of particles per film: $3.922 \cdot 10^{-3}$ g EC-DC per film $\Rightarrow$ # particles per film = $2.474 \cdot 10^{10}$

- Concentration of particles per film ($c_{particles}$): $1.100 \cdot 10^{11}$ particles cm^{-3}

Next, the transmittance of the films at 550 nm was determined using equation (1), where l_{film} is the thickness of the films (0.1 cm):

$$\%Tr_{550\ nm} = 100\ e^{-c_{particles}\ \sigma_{scattering}^{550\ nm}\ l_{film}} \qquad (1)$$

The following $\%Tr_{550\ nm}$ were obtained:

- $\%\ Tr_{550\ nm}^{25\ ºC} = 90.8\%$
- $\%\ Tr_{550\ nm}^{40\ ºC} = 0.2\%$

References

1 Vallan, L.; Otaegui, J. R.; Ruiz-Molina, D.; Hernando, J.; Roscini, C. Cost-Effective Paraffin Particles-Based Thermochromic Composites for Smart Windows and Greenhouses. *Small Struct.* **2025**, *6*, 2500250. https://doi.org/10.1002/sstr.202500250.

2 Otaegui, J. R.; Ruiz-Molina, D.; Hernando, J.; Roscini, C. Multistimuli-Responsive Smart Windows Based on Paraffin-Polymer Composites. *Chem. Eng. J.* **2023**, *463*, 142390. https://doi.org/10.1016/j.cej.2023.142390.

3 Li, X.-H.; Liu, C.; Feng, S.-P.; Fang, N. X. Broadband Light Management with Thermochromic Hydrogel Microparticles for Smart Windows. *Joule* **2019**, *3*, 290–302. https://doi.org/10.1016/j.joule.2018.10.019.

4 Zhou, Y.; Wang, S.; Peng, J.; Tan, Y.; Li, C.; Boey, F. Y. C.; Long, Y. Liquid Thermo-Responsive Smart Window Derived from Hydrogel. *Joule* **2020**, *4*, 2458–2474. https://doi.org/10.1016/j.joule.2020.09.001.

5 Zhou, Y.; Cai, Y.; Hu, X.; Long, Y. Temperature responsive hydrogel with ultra large solar modulation and high luminous transmission for smart window applications. *J. Mater. Chem. A*, **2014**, *2*, 13550-13555. https://doi.org/10.1039/C4TA02287D.

6 Zhang, Q.; Jiang, Y.; Chen, L.; Chen, W.; Li, J.; Cai, Y.; Ma, C.; Xu, W.; Lu, Y.; Jia, X.; Bao, Z. Ultra-Compliant and Tough Thermochromic Polymer for Self-Regulated Smart Windows. *Adv. Funct. Mater.* **202**1, *31*, 2100686. https://doi.org/10.1002/adfm.202100686.

7 Liang, X.; Guo, S.; Chen, M.; Li, C.; Wang, Q.; Zou, C.; Zhang, C.; Zhang, L.; Guo, S.; Yang, H. A temperature and electric field-responsive flexible smart film with full broadband optical modulation. *Mater. Horiz.* **2017**, *4*, 878–884. https://doi.org/10.1039/C7MH00224F.

8 Liang, X.; Chen, M.; Wang, Q.; Guo, S.; Zhang, L.; Yang, H. Active and passive modulation of solar light transmittance in a hybrid thermochromic soft-matter system for energy-saving smart window applications. *J. Mater. Chem. C* **2018**, *6*, 7054–7061. https://doi.org/10.1039/C8TC01274A.

9 Liu, Y.; Fan, J.; Plamthottam, R.; Gao, M.; Peng, Z.; Meng, Y.; He, M.; Wu, H.; Wang, Y.; Liu, T.; Zhang, C.; Pei, Q. Automatically Modulated Thermoresponsive Film

Based on a Phase-Changing Copolymer. *Chem. Mater.* **2021**, *33*, 18, 7232–7241. https://doi.org/10.1021/acs.chemmater.1c01389.

10 Ke, Y.; Balin, I.; Wang, N.; Lu, Q.; Tok, A. I. Y.; White, T. J.; Magdassi, S.; Abdulhalim, I.; Long, Y. Two-Dimensional SiO_2/VO_2 Photonic Crystals with Statically Visible and Dynamically Infrared Modulated for Smart Window Deployment. *ACS Appl. Mater. Interfaces* **2016**, *8*, 33112–33120. https://doi.org/10.1021/acsami.6b12175.

11 Zhu, J.; Zhou, Y.; Wang, B.; Zheng, J.; Ji, S.; Yao, H.; Luo, H.; Jin, P. Vanadium Dioxide Nanoparticle-based Thermochromic Smart Coating: High Luminous Transmittance, Excellent Solar Regulation Efficiency, and Near Room Temperature Phase Transition. *ACS Appl. Mater. Interfaces* **2015**, *7*, 27796–27803. https://doi.org/10.1021/acsami.5b09011.

12 Zheng, J.; Bao, S.; Jin, P. TiO_2(R)/VO_2(M)/TiO_2(A) Multilayer Film as Smart Window: Combination of Energy-Saving, Antifogging, and Self-Cleaning Functions. *Nano Energy* **2015**, *11*, 136–145. https://doi.org/10.1016/j.nanoen.2014.09.023.

13 Shen, N.; Chen, S.; Chen, Z.; Liu, X.; Cao, C.; Dong, B.; Luo, H.; Liu, J.; Gao, Y. The synthesis and performance of Zr-doped and W–Zr-codoped VO_2 nanoparticles and derived flexible foils. *J. Mater. Chem. A* **2014**, *2*, 15087–15098. https://doi.org/10.1039/C4TA02880E.

14 Zhu, J.; Huang, A.; Ma, H.; Chen, Y.; Zhang, S.; Ji, S.; Bao, S.; Jin, P. Hybrid films of VO2 nanoparticles and a nickel(ii)-based ligand exchange thermochromic system: excellent optical performance with a temperature responsive colour change. *New J. Chem.* **2017**, *41*, 830-835. https://doi.org/10.1039/C6NJ03369E.

15 Zhu, J. T.; Huang, A. B.; Ma, H. B.; Bao, S. H.; Ji, S. D.; Jin, P. Solar-thermochromism of a hybrid film of VO_2 nanoparticles and Co^{II}–Br–TMP complexes. *RSC Adv.* **2016**, *6*, 67396-67399. https://doi.org/10.1039/C6RA14232J.

16 Zhang, L.; Xia, F.; Yao, J.; Zhu, T.; Xia, H.; Yang, G.; Liu, B.; Gao, Y. Facile synthesis, formation mechanism and thermochromic properties of W-doped VO_2(M) nanoparticles for smart window applications. *J. Mater. Chem. C* **2020**, *8*, 13396–13405. https://doi.org/10.1039/D0TC03436C.

17 Wang, L. L.; Li, Z. S.; Cao, C. C.; Yang, J. L.; Yang, C.; Cao, X. Facile and dynamic infrared modulation of durable VO_2/CuI films for smart window applications. *Chem. Eng. J.* **2024**, *488*, 150972. https://doi.org/10.1016/j.cej.2024.150972.

18 Shen, N.; Chen, S.; Wang, W. J.; Shi, R.; Chen, P. C.; Kong, D. J.; Liang, Y. X.; Amini, A.; Wang, J. B.; Cheng, C. Joule heating driven infrared switching in flexible VO_2 nanoparticle films with reduced energy consumption for smart windows. *J. Mater. Chem. A* **2019**, *7*, 4516–4524. https://doi.org/10.1039/C8TA11071A.

19 Mukherjee, P. K. Phase Transitions among the Rotator Phases of the Normal Alkanes: A Review. *Phys. Rep.* **2015**, *588*, 1–54. https://doi.org/10.1016/j.physrep.2015.05.005.

20 Karimi, V.; Babicheva, V. E. Mie Calculations of Single Nanosphere Cross-Sections. **2021**, https://nanohub.org/resources/extcs. https://doi.org/10.21981/7P39-NK61.
